# Health service utilization after terrorism: a longitudinal study of survivors of the 2011 Utøya attack in Norway

**DOI:** 10.1186/s12913-015-0811-6

**Published:** 2015-04-15

**Authors:** Lise Eilin Stene, Grete Dyb

**Affiliations:** Norwegian Centre for Violence and Traumatic Stress Studies, NKVTS, Gullhaugveien 1-3, NO-0484 Oslo, Norway; Institute of Clinical Medicine, Faculty of Medicine, University of Oslo, Oslo, Norway

**Keywords:** Disaster medicine, Health services, Mental health services, Adolescent medicine, Stress disorders, Post-traumatic, Health services research, Needs assessment, Delivery of health care

## Abstract

**Background:**

For effective organization of health services after terror attacks, it is vital to gain insight into survivors’ health service utilization. Following the 2011 Utøya mass shooting in Norway, a proactive outreach programme was launched to prevent unmet help needs. All survivors received health services during the first five months, yet an important minority were not proactively followed-up. This study assessed the prevalence of health service utilization and factors associated with mental health service utilization among the survivors 5–15 months after the attack.

**Methods:**

The study comprised data from interviews using standardised questionnaires performed 4–5 (T1) and 14–15 (T2) months after the attack. Altogether 281 of 490 (57.3%) survivors answered questions on health service utilization at T2 and were included in this study. Users and non-users of mental health services were compared using Pearson Chi Square tests (categorical variables) and independent t-tests (continuous variables). Multivariate logistic regression analyses were conducted to examine the relationship between mental health service utilization at T2 and early (model 1) and concurrent (model 2) posttraumatic stress reactions, mental distress and somatic symptoms. Both models were adjusted for age, gender and predisaster utilization of mental health services.

**Results:**

Altogether 267 (95.0%) of 281 survivors reported contact with health services at T2, including 254 (90.4%) with ≥1 types of primary care services; and 192 (68.3%) with mental health services. In bivariate analyses, mental health service utilization was associated with female gender, injuries, PTSD, mental distress, somatic symptoms, and sleep problems. After multivariate adjustments for early symptom levels (model 1), only mental distress remained significantly associated with mental health service utilization at T2 (OR 2.8, 95% CI 1.2-6.8). In the analysis adjusting for concurrent symptom levels (model 2), only somatic symptoms were associated with mental health service utilization (OR 4.4, 95% CI 1.8-10.8).

**Conclusions:**

The high utilization of both primary and secondary health services among young survivors 5–15 months after the attack underscores the importance of allocating resources to meet the increased demand for services over a longer time period. The results further highlight the need to address somatic symptoms in disaster survivors who receive mental health services.

**Electronic supplementary material:**

The online version of this article (doi:10.1186/s12913-015-0811-6) contains supplementary material, which is available to authorized users.

## Background

A terrorist attack is a major public health challenge due to its unforeseen and devastating effects. It can cause many fatalities and induce severe physical and mental health problems among survivors. Acute traumatic stress symptoms are common and often attenuate within weeks, yet some survivors develop persistent mental health problems [1,2]. Posttraumatic stress disorder (PTSD) and depression are the most prevalent mental disorders after terrorist attacks [2,3]. Young survivors are particularly vulnerable as posttraumatic distress may impair their psychosocial development and academic attainment with potential long-term consequences [4]. Hence they may need long-term follow-up both in primary and secondary care. Unfortunately, large unmet mental health needs have been documented after terrorist attacks [5,6]. However, prior research is sparse, and primarily includes cross-sectional studies confined to mental health service (MHS) utilization in adult disaster survivors [7,8]. Since terrorist attacks frequently involve children and adolescents, [7,9,10] more research is needed on utilization of different types of health services in young survivors, including their need of long-term health care.

This study covered health care utilization data from two waves of a longitudinal survey of survivors of the mass shooting at the Utøya youth camp. On July 22, 2011, a single perpetrator committed two terrorist attacks in Norway. Following a bomb explosion in the Oslo Government Quarter, a shooting massacre was inflicted on the summer camp of the Norwegian Labour Party’s youth organisation on the Utøya islet outside Oslo. Overall 564 persons were isolated on the islet during the 1.5 h long shooting; 69 were killed; a large number were injured; and many tried to escape by swimming at the risk of drowning. The Utøya shooting is considered a severe trauma due to the scope of fatalities and injuries, the young age of those affected, the fact that they were designated targets and because many lost their close ones [4,11]. In the wake of the shooting, a new public health program was introduced to address the survivors’ psychosocial needs. In order to prevent the unmet help needs reported after previous terror attacks, [5,6] an early and proactive outreach programme was modelled to identify and provide treatment to survivors who would develop mental health problems. The survivors came from rural and urban municipalities in all parts of the country. The Norwegian Directorate of Health outlined a primary care based outreach with further referral to specialized MHS if needed. Municipal crisis teams were assumed to immediately contact all survivors and their families. It was further recommended that all survivors be assigned a municipal contact person, who was expected to continue follow-up for at least one year to ensure that survivors who developed mental health problems received treatment. The follow-up was anticipated to include three standardized screening assessments at 5–6 weeks, three months and one year after the attack [12]. Previous findings showed that a large majority of survivors were proactively contacted by municipal health services in the first five months after the attack [13]. Yet it is unknown if the proactive outreach was maintained throughout a year as recommended, and the extent of long-term utilization of MHS should also be determined.

The overall aim of this study was to contribute knowledge that may optimize the public health response to future terrorist attacks and disasters. Our specific objectives were to (a) report the prevalence of different types of primary care and specialized mental health services utilization; and (b) assess factors associated with mental health services utilization among survivors of terrorism in high income settings 5–15 months after the attack.

## Method

### Participants and design

The police identified 495 survivors who had been on Utøya islet during the shooting. Postal study invitations were sent to 490 survivors; we excluded four children aged <13 years and one survivor living abroad. Semi- structured face-to-face interviews were performed by trained clinicians at 4–5 months (T1) and 14–15 months (T2) after the attack. All survivors (n = 490) were invited at both waves. The average post-disaster time of interview within T1 assessments was 4 months and 3 days (95% Confidence Interval 14 months and 1 day −14 months and 4 days); within T2 assessments it was 14 months and 22 days (95% CI 14 months and 19 days - 14 months and 25 days). At the end of the interview participants filled out a questionnaire covering e.g. sociodemographic data and health care utilization. Overall 325 (66%) survivors participated at T1; 285 (58%) participated at T2, while 255 (52%) participated at both T1 and T2. Survivors who participated at T2 did not differ from other survivors (i.e. non-participants and participants at T1 only) with respect to gender, age or residential region. Compared to survivors who participated at both waves, those who participated at T1 only were more likely to be non-Norwegian and to report higher exposure levels, while survivors who participated at T2 only reported higher levels of posttraumatic stress reactions, mental distress and somatic symptoms. No significant differences were found with respect to MHS utilization. Altogether 281 (57%) survivors answered at least one question on health service utilization at T2 and were included in this study. The study procedures have been further described previously [14].

### Ethics

Participants gave written informed consent. In accordance with Norwegian Laws, parental consent was requested for survivors younger than 16 years of age. The interviews were conducted by health practitioners who had received training in conducting research interviews in traumatised individuals at a 1-day seminary. The interviewers were instructed to offer assistance in contacting suitable services if they identified unmet help needs among survivors. The interviewers worked in teams of two, and after each survey wave there was a 1-day meeting for interviewers to share experiences. Furthermore, a phone line was provided for the interviewers where they could discuss challenges they met during interviews, and receive support. The Regional Committees for Medical and Health Research Ethics South East and North approved the study.

### Measures

#### Health services

The questionnaires covered contact with specialized MHS and primary care services, including the municipal crisis team, the contact person, the regular general practitioner (GP), and other primary care. While primary care services are organized at a municipal level, four Regional Health Authorities (RHAs) are responsible for the provision of specialized MHS to the population in their region. A crisis team of health care practitioners and other personnel (e.g. priest or social worker) should be available in all municipalities to provide acute psychosocial intervention during crisis. The contact person was expected to proactively follow up survivors and arrange three screening assessments throughout at least one year after the attack. The screening was intended to identify survivors’ help needs, and covered e.g. symptoms of PTSD, anxiety, depression, physical complaints, social support, and functioning at school/work. The profession of the contact person could vary according to the availability of resources in the municipality (e.g. municipal psychologist, psychiatric nurse, or social worker). Furthermore, all residents in Norway are entitled to have a regular GP, and more than 99% of the population do [15]. The regular GP maintains the general medical follow-up, and is ordinarily responsible for referrals to specialized health services. Other primary care included municipal help personnel; such as school nurse, school pedagogical & psychological service, and municipal psychologists. Contact with the crisis team was surveyed at T1 alone, since it provides only acute, and not long-term, psychosocial intervention in connection with disasters. Contact with the other health services was assessed both at T1 and T2. Except for the crisis team and the contact person at T1, we also assessed the consultation frequency and perceived usefulness, rated as none, some, and high. T1 covered contact with health services since the attack until T1 (ca. 0–5 months post-disaster); T2 covered such contact from January 1, 2012 until T2 (ca. 5–15 months post-disaster). Participants were additionally asked whether they currently received the help they needed; for instance from a psychologist, physician, nurse, social worker, or other professionals. Moreover, predisaster MHS utilization was assessed by a dichotomous question on whether or not they had received MHS before the terror attack (yes/no).

#### Health

Posttraumatic stress reactions in the preceding month were measured using the University of California at Los Angeles PTSD Reaction Index (UCLA PTSD-RI) [16]. The total score comprises 17 items that correspond to the 17 DSM–IV symptoms of PTSD rated on a 5-point Likert scale (range 0 = never to 4 = most of the time). Three items have two alternative formulations that are valuated by the item with the highest score. Therefore the instrument is overall composed of 20 items. Reactions experienced “much of the time” and “most of the time” were defined as clinical symptoms. Levels of PTSD were grouped by the diagnostic criteria of PTSD. Five items covered re-experiencing, seven covered avoidance/numbing and five covered hyperarousal. Participants meeting the criteria for three symptoms groups were probable PTSD cases, while partial PTSD implicated that the criteria were met for two symptom groups [17]. The mean scores of the 17 items were used in the multivariate analyses. Cronbach’s alpha was 0.89 both at T1 and T2. Mental distress was assessed with the Hopkins Symptom Checklist-8 (SCL-8) [18]. It contains eight items scored on a scale from 1 (not bothered) to 4 (very much bothered) which appraise symptoms of depression and anxiety the past two weeks. The mean score of five of the items can be dichotomised by a validated cut-off at >2.0 to serve as a measure of anxiety and depression [19]. The mean scores of all eight items were used in the multivariate analyses, with a Cronbach’s alpha 0.86 (T1) and 0.90 (T2). The short versions of the Hopkins Symptom Checklist have demonstrated high psychometric qualities in population-based studies [20]. Somatic symptoms the past two weeks were measured by a short version of Children’s Somatic Symptoms Inventory (CSSI-8) [21]. The eight items covered pain in stomach, head, lower back, and arms/legs, faintness/dizziness, rapid heartbeat, nausea/stomach problems, and weakness. Each item scored on a scale from 1 (not bothered) to 4 (very much bothered). The mean score was applied in the analyses; Cronbach’s alpha was 0.78 both at T1 and T2. Finally, survivors were asked how often during the last month they had trouble going to sleep or waking up often during the night using five response alternatives. Those who answered two times a week or more were classified as having sleep problems.

#### Other variables

Terror exposure was measured at T1 by a sum score of 13 potentially traumatic events occurring during the attack. This assessment was explicitly designed to cover critical events experienced at the island during the attack. It has previously been described and shown as independently associated with mental health problems [14]. We also assessed physical injuries that required medical assistance; and death of someone close (close friend, family member or boy-/girlfriend) during the attack. Perceived social support was measured by the mean score of seven items from the validated Duke-University of North Carolina Functional Social Support Questionnaire (FSSQ) scored on a scale from 1 (much less than I would like) to 5 (as much as I would like) [14,22]. Sociodemographic data included gender, age, country origin, financial status, and whether the survivor was living alone, lived in a peripheral municipality, or had relocated after the attack. Age in years was assessed both as a continuous variable with one decimal (multivariate analysis) and dichotomized according to the Norwegian age of minority into younger than 18 versus 18 years or older at the time of the attack. Survivors with both parents born abroad were defined as having non-Norwegian origin. Furthermore, survivors were asked how they perceived their own (survivors who did not live with parents) or parents’ (survivors who lived with parents) economy compared to others. There were five response alternatives which were dichotomized into financially disadvantaged (much or somewhat poorer) or not (similar, somewhat better, and much better). Peripheral home municipality described the location of the survivor’s home municipality at T1 in relation to communities of a certain size according to Statistics Norway’s classification of centrality [23]. Municipalities that were located more than 45 minutes’ travelling time from communities with at least 15000 inhabitants were defined as peripheral (Additional files 1 and 2).

### Statistical analysis

We applied Pearson Chi Square tests (categorical variables) and independent t-tests (continuous variables) to test group differences between survivors who used MHS and those who did not. The Fisher’s exact test was used for number of primary care services at T1 due to low number of expected count. Multivariate logistic regression analyses were performed using MHS utilization at T2 (yes/no) as outcome. Due to sample size considerations, we restricted the number of independent variables to six factors. The six variables were selected by an à priori approach based on factors considered as relevant in the guidelines for proactive follow-up after the attack. Two models were tested. Model 1 adjusted for age; gender; predisaster MHS utilization; and posttraumatic stress reactions, mental distress, and somatic symptoms measured at T1. Model 2 adjusted for age; gender; predisaster MHS utilization; and posttraumatic stress reactions, mental distress, and somatic symptoms measured at T2. The statistical inferences were based on a two-sided significance level of 0.05. We reported the crude and adjusted odds ratios (OR) and 95% confidence intervals (CI). The analyses were performed with IBM SPSS version 20.0.

## Results

**Figure 1:**
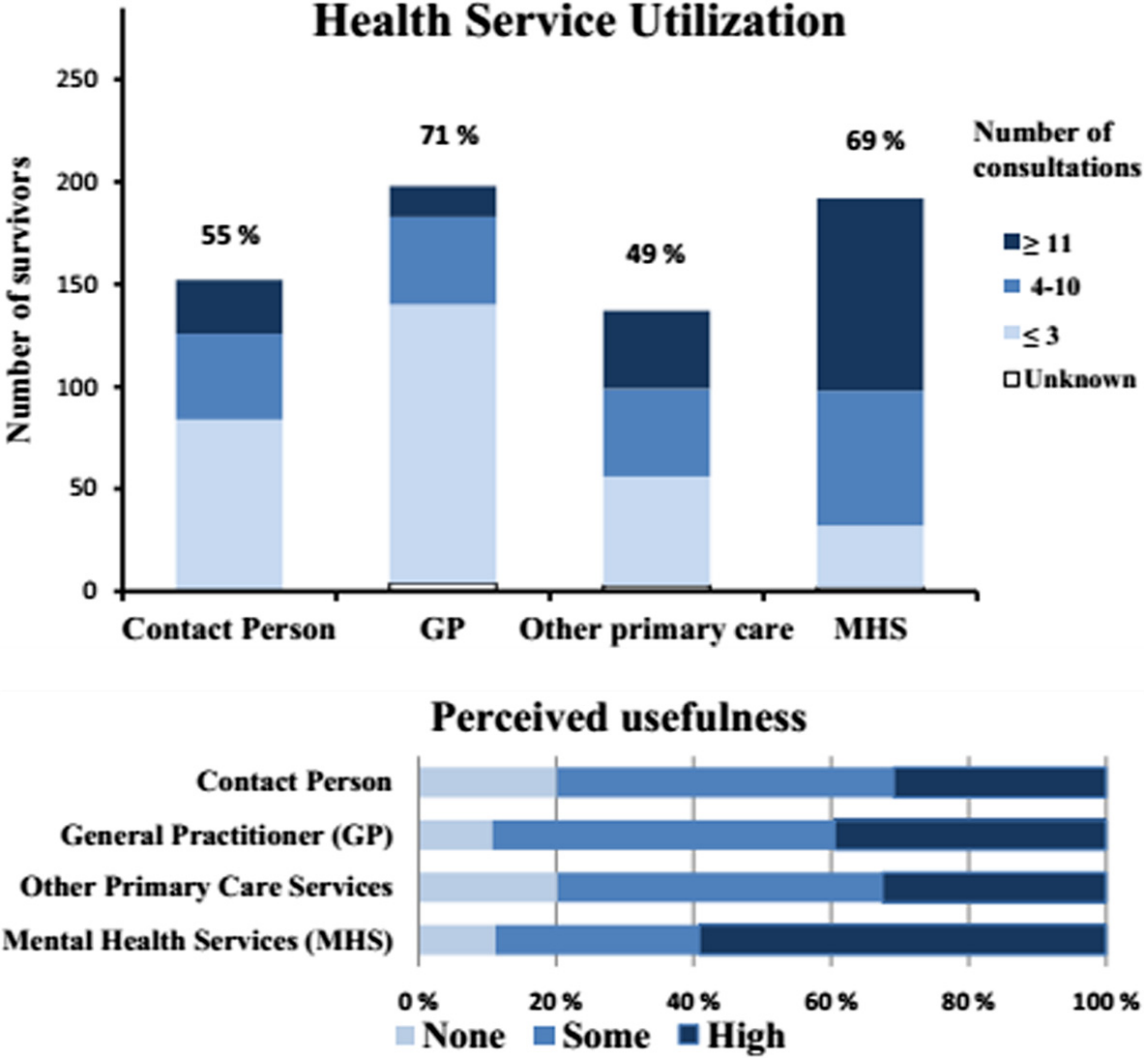
Health Service Utilization. Survivors’ health service utilization and self-perceived usefulness of different types of health services 5–15 months after the Utøya attack (n = 281).

Altogether 133 (47.3%) of 281 survivors were female; the median age was 18. 2 years (range 13. 3–46. 7), and the mean age 19. 3 years (sd 4.3). Figure 1 illustrates the prevalence and perceived usefulness of different types of care. At T2 267/281 (95.0%) survivors reported contact with any health services, including 254/281 (90.4%) with one or more types of primary health care services; and 192 survivors (68.6%) who used MHS. Five of the survivors who were not in contact with any health services had clinical levels of mental distress and/or full or partial PTSD. Table 1 shows the survivors’ characteristics by MHS utilization at T2. MHS utilization was associated with female gender and being injured. Otherwise no significant differences were found with respect to sociodemographic and disaster-related characteristics. The mean age was 19. 5 years (sd 4, 9) in MHS users and 19. 0 (sd 2. 6) in non-users (p = 0.320). MHS utilization was more likely among survivors with elevated levels of PTSD, mental distress, somatic symptoms, and sleep problems at T1 and T2. MHS utilization did not significantly differ with respect to social support or exposure. At T2 most survivors who did not use MHS had no contact with a designated contact person in 2012 (Table 2). MHS utilization was associated with contact with the regular GP; at T2 MHS utilization was also associated with contact with other types of primary care services. Unmet needs for help or predisaster MHS utilization did not significantly differ by MHS utilization. In the multivariate analysis adjusting for symptom levels at T1 (model 1), only mental distress remained significantly associated with MHS utilization at T2. In the multivariate analysis adjusting for symptom levels at T2 (model 2), only somatic symptoms was associated with MHS utilization (Table 3).

**Table 1:** **Characteristics of survivors by mental health service (MHS) utilization 5–15 months after the Utøya attack**

		**MHS utilization (n=280)**		
		**Yes (n = 192)**	**No (n = 88)**	
**Characteristics**		**n (%)**	**n (%)**	**p-value**
Female (n = 280)		99 (51.6)	33 (37.5)	0.029
Minor (<18 years) (n = 280)		93 (48.4)	35 (39.8)	0.177
Non-Norwegian origin (n = 274)		20 (10.6)	6 (7.0)	0.337
Living alone (n = 278)		43 (22.4)	24 (27.9)	0.321
Financially disadvantaged (n = 273)		39 (20.6)	16 (19.0)	0.763
Peripheral home municipality (n = 278)		27 (14.1)	10 (11.5)	0.548
Relocation 0–5 months after attack (n = 249)		20 (12.0)	13 (15.9)	0.396
Relocation 0–15 months after attack (n = 280)		43 (22.4)	28 (31.8)	0.092
Injured (n = 273)		46 (24.7)	10 (11.5)	0.012
Death of someone close (n = 280)		137 (71.4)	62 (70.5)	0.878
PTSD at T1 (n = 251)	Full	21 (12.4)	7 (8.5)	0.034
	Partial	64 (37.9)	20 (24.4)	
	No	84 (49.7)	55 (67.1)	
PTSD at T2 (n = 280)	Full	16 (8.3)	2 (2.3)	0.015
	Partial	40 (20.8)	10 (11.4)	
	No	136 (70.8)	76 (86.4)	
Mental distress	T1 (n = 251)	89 (52.7)	23 (28.0)	<0.001
	T2 (n = 280)	62 (32.3)	18 (20.5)	0.042
Sleep problems	T1 (n = 250)	81 (48.2)	18 (22.0)	<0.001
	T2 (n = 277)	59 (31.1)	14 (16.1)	0.009
**Continuous variables**		**Mean (sd)**	**Mean (sd)**	
Mean sum of exposure (0–13) (n = 275)		8.44 (2.29)	8.13 (2.12)	0.281
Social support	T1 (n = 251)	4.52 (0.61)	4.62 (0.47)	0.125
	T2 (n = 280)	4.54 (0.59)	4.58 (0.62)	0.677
Somatic symptoms	T1 (n = 251)	1.82 (0.58)	1.54 (0.43)	<0.001
	T2 (n = 280)	1.75 (0.54)	1.41 (0.37)	<0.001

**Table 2:** **The survivors’ health service utilization and unmet help needs by mental health service (MHS) utilization 5–15 months after the Utøya attack**

			**MHS utilization (n = 280)**		
			**Yes, n = 192 (%)**	**No, n = 88 (%)**	**p-value**
Crisis team	At T1 (n = 247)		143 (85.6)	75 (93.8)	0.064
Contact person	At T1 (n = 244)		136 (84.0)	67 (81.7)	0.658
	At T2 (n = 274)		112 (59.9)	40 (46.0)	0.031
Regular GP	At T1 (n = 247)		118 (70.7)	37 (46.2)	<0.001
	At T2 (n = 280)		151 (78.6)	47 (53.4)	<0.001
Other primary care	At T1 (n = 244)		105 (64.0)	51 (63.7)	0.967
	At T2 (n = 279)		108 (56.5)	29 (33.0)	<0.001
Unmet needs for help	At T1 (n = 242)		26 (16.0)	10 (12.5)	0.465
	At T2 (n = 267)		22 (12.1)	11 (12.9)	0.844
Number of primary care services	At T1 (n = 250)	0	3 (18)	0 (0.0)	0.036
		1	10 (6.0)	7 (8.5)	
		2	34 (20.2)	18 (22.0)	
		3	60 (35.7)	41 (50.0)	
		4	61 (36.3)	16 (19.5)	
	At T2 (n = 280)	0	13 (6.8)	13 (14.8)	<0.001
		1	44 (22.9)	41 (46.6)	
		2	78 (40.6)	27 (30.7)	
		3	57 (29.7)	7 (8.0)	
Predisaster MHS utilization	(n = 280)		56 (29.2)	20 (22.7)	0.261
MHS utilization at T1	(n = 246)		137 (82.5)	39 (48.8)	<0.001

**Table 3:** **Multivariate logistic regression analyses of survivor characteristics associated with mental health service utilization 5–15 months after the Utøya attack**

				**Model 1 (n = 251)**			**Model 2 (n = 280)**		
	**Crude OR**	**95% CI**	**p-value**	**Adjusted OR**	**95% CI**	**p-value**	**Adjusted OR**	**95% CI**	**p-value**
Female gender	1.77	(1.06-2.97)	0.029	1.26	(0.69-2.27)	0.452	1.16	(0.65-2.05)	0.615
Age	1.03	(0.96-1.09)	0.421	1.03	(0.96-1.11)	0.425	1.02	(0.95-1.09)	0.564
Predisaster MHS utilization	1.40	(0.78-2.52)	0.262	1.06	(0.55-2.06)	0.865	0.97	(0.52-1.84)	0.935
T1 PTSD (UCLA PTSD-RI)	2.49	(1.65-3.76)	<0.001	1.04	(0.48-2.25)	0.918			
T1 Mental distress (SCL-8)	3.37	(2.06-5.52)	<0.001	2.81	(1.16-6.78)	0.022			
T1 Somatic symptoms (CSSI-8)	2.95	(1.67-5.21)	<0.001	1.21	(0.56-2.63)	0.628			
T2 PTSD (UCLA PTSD-RI)	2.61	(1.68-4.05)	<0.001				1.82	(0.87-3.79)	0.112
T2 Mental distress (SCL-8)	2.35	(1.48-3.73)	<0.001				0.67	(0.29-1.53)	0.338
T2 Somatic symptoms (CSSI-8)	5.54	(2.79-10.99)	<0.001				4.35	(1.75-10.83)	0.002

Both models adjusted for gender, age and predisaster MHS utilization; in addition model 1 adjusted for PTSD symptoms, mental distress and somatic symptoms at T1; model 2 for PTSD symptoms, mental distress and somatic symptoms at T2. OR = Odds ratio, CI = Confidence Interval.

## Discussion

In the wake of the Utøya massacre, the survivors’ utilization of health services was extensive both immediately and at longer-term. The grand majority received one or more types of primary health services both directly after the attack and the following year. Most survivors used specialized mental health services (MHS) in addition to the primary care based outreach. MHS utilization in the post-acute phase was more common in survivors who were injured, and those with high levels of posttraumatic stress, mental distress, and somatic symptoms, but differed little by sociodemographic characteristics.

### Interpretation and comparison

The survivors’ high symptom levels and extensive utilization of MHS emphasize the severity of the attack. They further experienced an extended period of concern because of the trial and the high media coverage. The proactive outreach programme outlined that all survivors were assigned a contact person to organize screening assessments throughout at least a year. Yet one in six survivors reported that they did not have a contact person the first 4–5 months, while nearly half had no contact with the contact person 5–15 months after the attack. The high number of survivors without a contact person at T2 might represent a lack of continuity in the follow-up. Yet other health care practitioners might have performed the follow-up, as 95% of the survivors reported contact with at least one type of health services at T2. The recommendations were not mandatory; some municipalities may therefore not have provided contact persons. Furthermore, some survivors may have been proposed a contact person, and declined.

The utilization frequency and perceived usefulness was higher for MHS compared to primary care services. Since access to MHS usually occurs through referral from primary care services, survivors who received MHS may have had both a greater need for care and willingness to undergo therapy. Referrals to MHS often involve participation in an intervention lasting several weeks or months which requires higher consultation frequency. Due to proactive follow-up, the contact person and other primary care practitioners may have been more likely to interact with survivors who did not want or need health care. A previous study of the early proactive outreach after the Utøya attack found that survivors who did not use MHS the first 4–5 months were more likely to have had contact with the municipal crisis team or a contact person [13]. Contrarily, in the current paper survivors who did not use MHS the following year were less likely to have had concurrent contact with the contact person; more than half had no contact with the contact person (Table 2). This may be of concern, considering that post-disaster psychopathology, such as PTSD, may have a delayed onset of more than six months [24]. Besides, the survivors were at risk for potential retraumatization for a prolonged period due to the trial and the high media coverage [25,26]. Utilization of MHS was associated with utilization of the regular GP, which could be expected as the GP normally issues drug prescriptions, sick leaves, and referrals to MHS.

In contrast to prior findings, [8] MHS utilization differed little by sociodemographic background, except that MHS utilization was higher among female survivors. Contrarily, MHS utilization differed significantly by impaired mental and physical health. This may indicate that a proactive outreach promotes that health care delivery primarily is based on needs rather than sociodemographic factors. Most survivors with clinical levels of PTSD or mental distress received MHS. In comparison, a minority of young survivors with severe posttraumatic stress reactions received counseling after the 1995 Oklahoma City Bombing and September 9/11 attacks [5,6]. Yet in our study one in five survivors who did not receive MHS had clinical levels of mental distress, and 14% had full or partial PTSD (Table 1). Among those who did not receive MHS and had clinical levels of mental distress and/or full or partial PTSD, there were five survivors who were not in contact with primary care services between T1 and T2. Hence the majority were in contact with primary care services. This could suggest a failure at the primary health care level to identify and meet the needs for treatment in an important minority of survivors. Contrary to former findings, [5,27] survivors with prior experience with MHS were not more likely to receive MHS after the Utøya attack. The proactive outreach may have rendered MHS more accessible also for survivors without prior experiences with MHS, since they did not have to initiate contact with treatment services themselves. Nonetheless, the consultation frequency was higher among survivors with predisaster MHS utilization.

Many survivors moved away from home to start at university or college shortly after the attack. Relocation was considered a potential risk factor for posttraumatic distress, as it could involve a loss of support from established social networks and family. Relocation was also considered as a potential risk factor for disruption of existing care or failure to initiate treatment; however, MHS utilization did not significantly differ by relocation. This could suggest that the MHS follow-up was maintained despite relocation.

The multivariate analysis showed that symptoms of depression/anxiety at T1 remained significantly associated with MHS utilization at T2 after adjustments (model 1). It is therefore possible that early symptoms of depression/anxiety after disasters indicate a need for prolonged treatment. It is also possible that the initial therapy was more successful at identifying and treating posttraumatic stress reactions than depression and anxiety. When we adjusted for symptoms at T2, only somatic symptoms remained significantly associated with MHS utilization (model 2). One possible explanation could be that somatization is an arduous aftereffect of long-term mental health problems after disasters [28]. Furthermore, somatic ailments may have promoted counselling, and hence increased the likelihood for referral to MHS. The somatic symptoms could also be directly related to the terrorist attack, and potentially induce, maintain or worsen mental illness at long-term. Yet somatic symptoms remained significantly associated with MHS utilization when we added injuries to the independent variables in model 2 (data not shown). Somatic symptoms are associated with adverse psychosocial and academic consequences in adolescents [29]. It is therefore important that health care practitioners address somatic symptoms also in disaster-survivors who are not physically injured. Moreover, it has been shown that the prevalence and co-occurrence of somatic and psychological symptoms in low income settings are similar to those in high income settings [30]. Accordingly, the importance of assessing somatic symptoms in addition to psychological symptoms is likely to be pertinent also for low income settings.

A challenge with proactive outreach is to decide who should be included in the screening. The survivors in our study had been isolated on an islet, and may therefore have been easier to identify than survivors of other disasters. Yet the survivors were dispersed over a large number of municipalities in the entire country after the attack. The geographical dispersion disfavoured a centralized screen and treat response, which has recently showed promising results [31]. Our study indicates that a proactive outreach is feasible also when survivors are geographically dispersed. External validity may nonetheless be limited by differences in how healthcare provision is organised and financed. Norway is a country with universal healthcare, therefore access to specialized health care may depend less on personal economy than in countries with insurance-based healthcare. Furthermore, the study might not be representative for low-resource settings. A proactive outreach can be resource-demanding. A key challenge in low income settings is the limited availability of mental health resources. In accordance with recent WHO guidelines, access to mental health inventions could be improved by integrating the delivery of psychotherapy by non-specialists, such as community health workers in primary care [32]. A primary care based proactive outreach might thus be applicable also in low income settings. More research is needed to develop effective evidence-based outreach in low income settings, where the risk of disasters is highest [33].

The unpredictability and chaotic circumstances of terrorism make it challenging to execute studies. It is therefore important that researchers in advance plan how to implement studies after terror attacks. A linkage to registers could contribute objective and accurate measures of survivors’ health and service utilization before and after the attack, and thus enable a prospective design with a baseline assessment.

### Strengths and limitations

The current study contributes new insight into the long-term delivery of different types of health services to young survivors of terrorism. Prior research on disaster-related health service utilization is still limited, and has usually addressed adult survivors using a cross-sectional design, and not covered several services [7,8,34]. Our study included data from in-depth interviews at two time points after the attack. The longitudinal perspective is essential to increase our understanding of how initial reactions and receipt of health care are related to later MHS utilization, and thus optimize post-disaster health care delivery. Furthermore, the Utøya shooting was geographically constricted to a small island where all survivors were exposed to a life-threatening event and could be identified. Former studies have commonly lacked information about the number and identity of directly affected survivors, and consequently met difficulties with selecting a representative sample. The clear definition of our study population may therefore have yielded more reliable estimates. Yet the study had several limitations. Our study included 57% of the survivors, and selection bias may have occurred. Participation did not significantly differ by age, gender, or geographic region of residence, but survivors who participated in T1 only scored higher on exposure. Moreover, survivors who participated at T2 only had more severe posttraumatic stress reactions, mental distress, and somatic symptoms. This may imply that non-participation was associated with poorer mental and physical health. However, survivors who participated at one wave did not significantly differ from two-wave participants with respect to MHS utilization. Study participation might have influenced health service utilization and recovery, as interviewers may have assisted in acquiring treatment for participants with unmet needs for help. Furthermore, the study lacked predisaster data and was based on self-reports, which could be inaccurate. Survivors might also have used health services due to concerns that were not or only partially related to the terrorist attack. Another potential limitation was the variance in the time of interview. T1 covered health service utilization from the attack until T1, which was mainly effectuated in November and December 2011, while T2 covered health service utilization in 2012. Eight survivors completed T1 early in 2012, of whom three reported MHS utilization at T2. Hence there was a potential overlap between the T1 and T2 measurement of MHS utilization for three survivors. Due to sample size considerations, we had to limit the number of independent variables; our multivariate analyses might therefore have missed relevant confounders. Finally, our analysis might have missed significant differences due to a relatively small sample size (type II error).

## Conclusions

The study demonstrated a high utilization of primary and secondary health care among young survivors 5–15 months after the Utøya attack. It is therefore important to allocate enough resources to meet the increased demand for services after disasters, which may persist over a longer period of time. A larger proportion of survivors with severe posttraumatic distress received mental health services with the proactive outreach after the Utøya attack compared to terror attacks where health service provision depended on self-referral. Yet an important minority with clinical levels of PTSD or mental distress did not receive mental health services, hence further improvement in outreach models is required. The findings also underscore the need to address somatic symptoms among survivors who receive mental health services.

## Additional files

Additional file 1:
**Interview questionnaire at T1 (in Norwegian).**
Additional file 2:
**Interview questionnaire at T2 (in Norwegian).**
