# Supplementary material for: Health service utilization after terrorism: a longitudinal study of survivors of the 2011 Utøya attack in Norway
Source: BMC Health Serv Res. 2015 Apr 15;15:158. doi: 10.1186/s12913-015-0811-6 (PMC4457986; doi:10.1186/s12913-015-0811-6)
Supplement: Additional file 1: — Interview questionnaire at T1 (in Norwegian). [file 12913_2015_811_MOESM1_ESM.pdf]

Terrorangrepet 22. juli 2011.

Opplevelser og reaksjoner hos de som var på Utøya

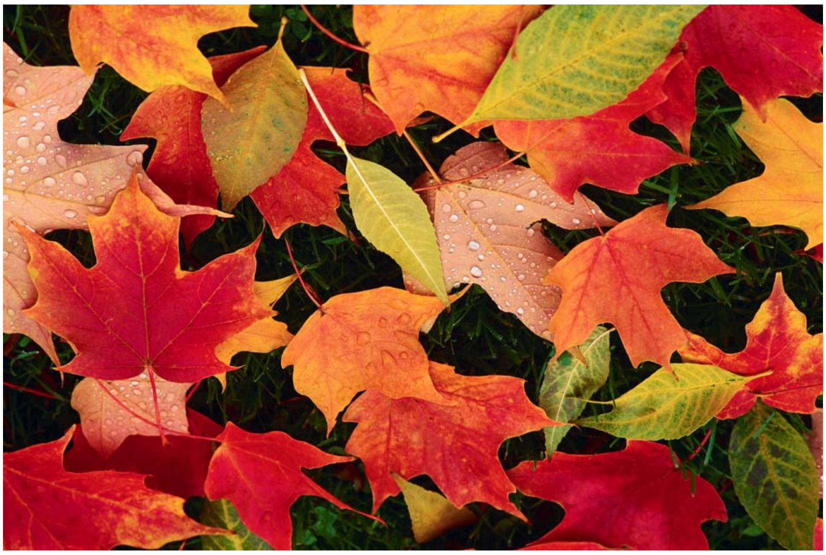

Nasjonalt kunnskapssenter
om vold og traumatisk stress

Instruks til intervjuer

Først skal SAMTYKKE ERKLÆRINGEN underskrives av informanten. Ha alltid den med til intervjuene sammen med et eksemplar av informasjonsskrivet. Informasjonsskrivet som er sendt ut beskriver studien og gjennomføringen av denne. Informantene kan trekke seg også underveis i intervjuet.

Fyll ut arket med INFORMASJON OM INTERVJUET OG KONTAKTINFORMASJON før du leser introduksjonen og slår på diktafonen.

STILL ALLE SPØRSMÅL – ikke hopp over spørsmål du synes at de allerede har besvart. Temaer kan være overlappende, men spørsmålene skal likevel stilles. Dersom informanten ikke forstår spørsmålet: gjenta først spørsmålet langsommere. Deretter kan du forklare ORD i spørsmålet, og hva de betyr. Prøv å unngå og utdype spørsmålet med egen fortolkning.

Introduksjon

(Les denne introduksjonen sammen med ungdommen før du starter intervjuet)

Vi kommer til å snakke med mange ungdommer som overlevde terroren på Utøya og foreldrene deres. Grunnen til det er at det er viktig at vi lærer mer om hvilke konsekvenser slike handlinger kan ha, og hva som kan bidra til å minske belastningene etter slike opplevelser.

Jeg er opptatt av å høre hvordan det var for deg.

Det du forteller meg blir ikke fortalt videre til foreldrene dine, på samme måte som du ikke får vite fra oss hva foreldrene dine forteller. Hvis du sier noe som gjør at jeg blir bekymret for deg må vi drøfte hva vi kan gjøre for å hjelpe deg videre. Dersom du er under 18 år kan det hende jeg må snakke med foreldrene dine om dette, men da vil jeg si ifra til deg først.

Si ifra til meg hvis det er noe du ikke forstår, eller hvis det er noe du synes det er vanskelig å svare på. Hvis du vil ha en pause så er det helt i orden. Dersom det er spørsmål du ikke ønsker å svare på, sier du fra om det underveis. Du trenger ikke å forklare hvorfor du ikke ønsker å svare på spørsmål.

Først spør jeg deg om din historie fra hendelsen. Etter det vil jeg stille en del spørsmål med faste svaralternativ. Til slutt oppsummerer vi sammen.

Jeg har med en diktafon for at vi etter intervjuet kan skrive ned det du forteller. Er det i orden for deg?

(Sett på diktafonen)

	Prosjekt	**1049371**
	Skjemanummer	

ID **NB! Skriv inn skjemanummer (id.nr.) og les det høyt opp på begynnelsen av intervjuet.**

Id-nr. 1

Narrativ

Kronologisk historie

Jeg vet at du var på Utøya under terrorangrepet. Fortell meg om det. . . .

Dersom informanten ikke svarer på det kan man si: **Fortell meg om hvor du var da gjerningsmannen kom til Utøya?**

La informanten fortelle mest mulig fritt.

Hjelp informanten videre ved å si: **Hva skjedde da?**

Hjelp informanten til å utdype ved å si: **Fortell meg om det. Eller gjenta det siste de sa.**

Det verste

Nå har du fortalt meg hva som skjedde.

Av alt det som skjedde, hva synes du var det verste som du opplevde?

Beslutningsprosesser

Da det skjedde, tenkte du eller sa du noe til deg selv for at du skulle føle deg bedre? Fortell

Når du tenker tilbake på alt det som skjedde, tenker du noen gang at du skulle gjort noe annerledes?

20©11 Synovate

Q1 **Hva opplevde du?**

Instruksjon, les opp:

Nå har du fortalt meg om dine opplevelser fra Utøya 22. juli. Nå stiller jeg deg noen detaljerte spørsmål om hva du opplevde. På disse spørsmålene trenger du bare å svare Ja eller Nei.

	Ja 1	Nei 2	
• Fikk du vite om bombingen i Oslo før skytingen på Utøya?	☐	☐	1
• Var du bekymret for noen i familien eller en venn pga bombingen i Oslo?	☐	☐	2
• Var du på Utøya da gjerningsmannen skjøt?	☐	☐	3
• Så du gjerningsmannen eller hørte du stemmen hans?	☐	☐	4
• Flyktet du fra gjerningsmannen (prøvde å gjemme deg eller løpe unna)?	☐	☐	5
• Hørte du skudd?	☐	☐	6
• Hørte du skrik?	☐	☐	7
• Kjente du lukt fra skytingen eller ble du oppmerksom på annen lukt?	☐	☐	8
• Var du redd for at du kom til å bli alvorlig skadet?	☐	☐	9
• Ble du fysisk skadet og hadde behov for medisinsk hjelp?	☐	☐	10
• Var du redd for at du kom til å dø?	☐	☐	11
• Så du at gjerningsmannen rettet pistolen mot deg eller oppdaget du at han skjøt etter deg?	☐	☐	12
• Så du noen bli skadet eller drept?	☐	☐	13
• Hørte du noen bli skadet eller drept?	☐	☐	14
• Hjalp du noen som ble alvorlig skadet?	☐	☐	15
• Var du i berøring med døde eller skadde under angrepet?	☐	☐	16
• Var du redd for at du skulle drukne?	☐	☐	17
• La du på svøm?	☐	☐	18
• Følte du deg truet av politiet (ikke gjerningsmannen)	☐	☐	19
• Så du noen som var døde?	☐	☐	20

Reaksjoner under hendelsen

Q2 Instruksjon, les opp:

De spørsmålene som jeg skal stille nå handler om reaksjoner du kan ha hatt under eller rett etter hendelsen.
I hvor stor grad..

	Ikke i det hele tatt 0	Litt 1	Noe 2	Mye 3	Veldig mye 4	
• Var du redd, eller tenkte du at dette var noe av det mest skremmende du har opplevd?	☐	☐	☐	☐	☐	1
• Følte du at du ikke kunne stoppe det som skjedde, eller at du trengte hjelp fra noen?	☐	☐	☐	☐	☐	2
• Var du veldig forvirret?	☐	☐	☐	☐	☐	3
• Følte du at det som skjedde, på en måte var uvirkelig, som om det skjedde i en film, og ikke i virkeligheten?	☐	☐	☐	☐	☐	4
• Følte du at det du opplevde, var motbydelig eller ekkelt?	☐	☐	☐	☐	☐	5
• Hadde du hjertebank?	☐	☐	☐	☐	☐	6
• Var du andpusten?	☐	☐	☐	☐	☐	7
• I hvilken grad opplevde du deg selv som følelsesmessig lammet, eller som om du var ved siden av deg selv?	☐	☐	☐	☐	☐	8

Mestringsstrategier

Q3 Instruksjon, les opp:

Nå skal jeg stille deg noen spørsmål om ting man kan si eller gjøre for å hjelpe seg selv når alvorlige ting skjer. Det er ikke noen riktige eller gale måter å reagere på. Bare tenk på hva du gjorde eller sa til deg selv for å få det bedre under terrorangrepet.

	Aldri 1	Noen ganger 2	Ofte 3	Meste-parten av tiden 4	
• Jeg tenkte over hvilke muligheter jeg hadde før jeg handlet.	☐	☐	☐	☐	1
• Jeg tenkte på de gode tingene i livet mitt	☐	☐	☐	☐	2
• Jeg gjorde noe for å forsøke å bedre min situasjon	☐	☐	☐	☐	3
• Jeg prøvde å ikke tenke på det som hendte	☐	☐	☐	☐	4
• Jeg sa til meg selv at det ville gå bra	☐	☐	☐	☐	5
• Jeg sa til meg selv at uansett hva som skjer, så kan jeg håndtere det	☐	☐	☐	☐	6
• Jeg fantaserte om at alt var i orden	☐	☐	☐	☐	7
• Jeg ba Gud hjelpe meg	☐	☐	☐	☐	8
• Jeg hadde kontakt med andre på øya for å finne ut av hva som var lurt å gjøre	☐	☐	☐	☐	9
• Jeg søkte trøst hos noen	☐	☐	☐	☐	10
• Jeg holdt meg sammen med andre	☐	☐	☐	☐	11
• Jeg holdt kontakt med noen som ikke var på øya via sms eller telefon	☐	☐	☐	☐	12
• Jeg tenkte på hvorfor dette skjer	☐	☐	☐	☐	13

Egne skader

INSTRUKSJON: SPØRSMÅLENE OM SKADE SKAL KUN STILLES TIL DE SOM HAR SVART "JA" PÅ SPØRSMÅLET OM DE BLE FYSISK SKADET PÅ SIDE 2 **svar fra Q1.A.10**

Q4 **Du har fortalt meg at du ble fysisk skadet på Utøya. Jeg vil nå spørre deg om skadene du fikk og hva slags hjelp du har fått.**

Fikk du relativt enkle eller overfladiske skader, som for eksempel overfladiske kutt, skrubbsår eller forstuvning?

Ja ☐ 1
Nei ☐ 2

Q5 **Fikk du dypere eller mer alvorlige skader, eksempelvis i forbindelse med nærdrukning, dype kutt, skuddskader eller brudd?**

Ja ☐ 1
Nei ☐ 2

Q6 **Hvis dypere/mer alvorlige skader, hvor ble du skadet?**

Besvar alle punktene

	Ja 1	Nei 2	
• Hode	☐	☐	1
• Ansikt	☐	☐	2
• Bryst	☐	☐	3
• Rygg	☐	☐	4
• Mage	☐	☐	5
• Armer	☐	☐	6
• Ben	☐	☐	7
• Annet	☐	☐	8

20©11 Synovate

Q7 **Hvis annet, vennligst spesifiser:**

Annet, notér ____________________

Q8 **Medførte skadene at du ble bevisstløs?**

Ja .... ☐ 1
Nei .... ☐ 2

Q9 **Hvis du var ved bevissthet:**

Hvor smertepåvirket var du av skadene under hendelsen?

Svar på en skala fra 0 til 10, der 0 = ingen smerte og 10 = den mest ekstreme smerteopplevelsen du kan tenke deg

	00	01	02	03	04	05	06	07	08	09	10	
... .	☐	☐	☐	☐	☐	☐	☐	☐	☐	☐	☐	1

Q10 **Ble du behandlet for skader på legevakt?**

Ja .... ☐ 1
Nei .... ☐ 2

Q11 **Ble du innlagt i sykehus?**

Ja .... ☐ 1
Nei .... ☐ 2

HVIS NEI, GÅ TIL **q17**

Q12 **Hvilket sykehus?**

Ullevål .... ☐ 1
Drammen .... ☐ 2
Hønefoss .... ☐ 3
Bærum .... ☐ 4

Q13 **I tilfelle, hvor mange dager var du innlagt?**

Noter antall dager ☐☐☐ 1

Q14 **Ble du utskrevet fra sykehus til hjemmet?**

Ja .... ☐ 1
Nei .... ☐ 2

Q15 **Ble du overført til rehabilitering eller annen opptrening (døgnbehandling) før du kom hjem?**

Ja .... ☐ 1
Nei .... ☐ 2

HVIS JA PÅ Q15

Q16 **Hvor mange dager totalt?**

Noter antall dager ☐☐☐ 1

Q17 **Ble du behandlet for skader av annen lege eller helsepersonell?**

Ja .... ☐ 1
Nei .... ☐ 2

Q18 **Har du synlige arr etter skade?**

- Ja ☐ 1
- Nei ☐ 2

Q19 **Gir skaden(e) smerter i dag?**

- Ja ☐ 1
- Nei ☐ 2

Q20 **Har skaden endret utseendet ditt?**

- Ja ☐ 1
- Nei ☐ 2

Q21 **Har du fått fysiske skader som hemmer deg i det daglige? (for eksempel i aktiviteter/fritidsaktiviteter/skolefungering/daglige rutiner?)**

- Ja ☐ 1
- Nei ☐ 2

Q22 **Har skaden gitt deg andre plager?**

- Ja ☐ 1
- Nei ☐ 2

HVIS JA

Q23 **Hvilke plager?**

Noter ______________________________

____

____

____

____

____

Q24 **Hvor plaget er du av skaden(e) nå?**

- Ikke i det hele tatt ☐ 1
- I liten grad ☐ 2
- Noe ☐ 3
- I stor grad ☐ 4
- I veldig stor grad ☐ 5

Familie og venner: Tap og skader.

Q25 **Ble nær venn, kjæreste eller familiemedlem drept i terrorangrepet?**

- Ja ☐ 1
- Nei ☐ 2

HVIS JA

Q26 **Hvor?**

	Ja 1	Nei 2	
På Utøya	☐	☐	1
I regjeringskvartalet	☐	☐	2

HVIS JA

Q27 **Hvem?**

Velg en kategori for hver av de drepte nære

	Ja 1	Nei 2	
• Nær venn	☐	☐	1
• Kjæreste/partner	☐	☐	2
• Søsken	☐	☐	3
• Forelder	☐	☐	4
• Annen familie	☐	☐	5

Q28 **Ble nær venn, kjæreste eller familiemedlem skadet i terrorangrepet? (Slik at de ble behandlet i sykehus)**

- Ja ☐ 1
- Nei ☐ 2

20©11 Synovate

HVIS JA

Q29 **Hvor?**

	Ja 1	Nei 2	
På Utøya	☐	☐	1
I regjeringskvartalet	☐	☐	2

HVIS JA

Q30 **Hvem?**

	Ja 1	Nei 2	
• Nær venn	☐	☐	1
• Kjæreste/partner	☐	☐	2
• Søsken	☐	☐	3
• Forelder	☐	☐	4
• Annen familie	☐	☐	5

Q31 **Ble noen ungdommer fra fylkeslaget ditt i AUF drept?**

Ja ☐ 1
Nei ☐ 2
Ikke aktuelt ☐ 3

Sikkerhet og gjenforening

(Hjelp til intervjuer: Skytingen startet kl 17.22, Beredskapstroppen pågrep gjerningsmann kl 18.27.)

Q35 **Når kom du i sikkerhet etter skytingen på Utøya? Ca kl.**

Noter klokkeslett ☐☐/☐☐ 1

HVIS JA

Q32 **Hvis ja, angi hvor mange:**

Noter antall. Vet ikke antall = ubesvart ☐☐☐ 1

Q33 **Ble noen ungdommer fra fylkeslaget ditt i AUF skadet? (Slik at de ble behandlet i sykehus)**

Ja ☐ 1
Nei ☐ 2
Ikke aktuelt ☐ 3

HVIS JA

Q34 **Angi hvor mange**

Noter antall. Vet ikke antall = ubesvart ☐☐☐ 1

Q36 **Når ble du gjenforent med en av dine nærmeste? (den første du ble gjenforent med av nær familie/partner/kjæreste)**

Fredag ca kl. ☐☐/☐☐ 1
Lørdag ca kl. ☐☐/☐☐ 2
Senere ☐☐/☐☐ 3

Reaksjoner den siste måneden

Posttraumatiske stress reaksjoner

Instruksjon som leses opp:

Nå har vi snakket om hendelsene og hva du opplevde under terrorangrepet på Utøya og like etterpå.Man kan få ulike reaksjoner på dramatiske hendelser. Nå kommer jeg til å lese opp noen påstander om mulige reaksjoner etter terrorangrepet på Utøya 22. juli. For hver påstand vil jeg spørre hvor ofte du har opplevd å ha det slik DEN SISTE MÅNEDEN.
Her er et skjema som er til hjelp når du skal svare på hvor ofte du har opplevd problemet den siste måneden.

Ta frem frekvensarket og legg på bordet mellom dere. Gå gjennom de ulike alternativene før dere starter på listen.

Q37 **Hvor mye av tiden den siste måneden**

	Aldri<br>1	Sjelden<br>2	Noen ganger<br>3	Ofte<br>4	Nesten alltid<br>5	
• D4 Jeg er på vakt for fare eller ting jeg er redd for.	☐	☐	☐	☐	☐	1
• B4 Når noe minner meg om det som skjedde på Utøya 22. juli, blir jeg veldig ute av meg, redd eller trist.	☐	☐	☐	☐	☐	2
• B1 Jeg får skremmende tanker, ser for meg bilder eller hører lyder fra det som skjedde på Utøya 22. juli, selv om jeg ikke vil det.	☐	☐	☐	☐	☐	3
• D2 Jeg føler meg sur, sint eller rasende.	☐	☐	☐	☐	☐	4
• B2 Jeg drømmer om det som skjedde på Utøya 22. juli eller har mareritt om andre ting.	☐	☐	☐	☐	☐	5
• B3 Jeg føler at jeg er tilbake til da det ubehagelige skjedde på Utøya 22. juli, og opplever det om igjen.	☐	☐	☐	☐	☐	6
• C4 Jeg har lyst til å være alene og ikke sammen med venner.	☐	☐	☐	☐	☐	7
• C5 Jeg føler meg alene inni meg og føler ikke nærhet til andre mennesker.	☐	☐	☐	☐	☐	8
• C1 Jeg forsøker å ikke snakke om det som skjedde på Utøya 22. juli, tenke på det eller ha følelser rundt det.	☐	☐	☐	☐	☐	9
• C6 Jeg har problemer med å føle glede eller kjærlighet.	☐	☐	☐	☐	☐	10
• C6 Jeg har problemer med å føle tristhet eller sinne.	☐	☐	☐	☐	☐	11
• D5 Jeg blir lett urolig eller skvetter lett, for eksempel når jeg hører høye lyder eller når noe overrasker meg.	☐	☐	☐	☐	☐	12
• D1 Jeg har problemer med å få sove eller jeg våkner ofte om natten.	☐	☐	☐	☐	☐	13
• AF Jeg bebreider meg selv for noe av det som skjedde på Utøya 22. juli	☐	☐	☐	☐	☐	14
• C3 Jeg har problemer med å huske viktige ting fra det som skjedde på Utøya 22.juli.	☐	☐	☐	☐	☐	15
• D3 Jeg har problemer med å konsentrere meg eller være oppmerksom.	☐	☐	☐	☐	☐	16
• C2 Jeg forsøker å holde meg unna folk, steder eller ting som minner meg om det som skjedde på Utøya 22.juli.	☐	☐	☐	☐	☐	17

20©11 Synovate

Hvor mye av tiden den siste måneden

	Aldri	Sjelden	Noen ganger	Ofte	Nesten alltid	
• B5 Når noe minner meg om det som skjedde på Utøya 22. juli, får jeg sterke følelser i kroppen, for eksempel hjertebank, hodepine eller mageknip.	☐	☐	☐	☐	☐	18
• C7 Jeg tror ikke at jeg kommer til å leve lenge.	☐	☐	☐	☐	☐	19
• D2 Jeg krangler eller slåss mye	☐	☐	☐	☐	☐	20
• C7 Jeg er negativ eller pessimistisk når jeg tenker på fremtiden	☐	☐	☐	☐	☐	21
• AF Jeg er redd for at noe lignende som det som skjedde på Utøya 22. juli skal skje igjen.	☐	☐	☐	☐	☐	22
• Nye D3 Jeg er sint på noen andre for at de ikke gjorde mer for å forhindre det som skjedde på Utøya 22. juli, eller for at de ikke har gjort nok etterpå (gjelder ikke sinne mot gjerningsmannen).	☐	☐	☐	☐	☐	23
• Nye D2 Jeg tenker at andre vonde ting vil skje med meg i fremtiden.	☐	☐	☐	☐	☐	24
• Nye E2 Jeg gjør ting som skader eller kan skade meg selv eller andre. (For eksempel å kjøre for fort, bruke narkotika, drikke veldig mye, skade meg selv eller gjøre andre farlige ting).	☐	☐	☐	☐	☐	25
• Nye D3 Jeg klandrer meg selv for at jeg ikke er blitt fortere bra.	☐	☐	☐	☐	☐	26
• Nye D4 Jeg skammer meg over noe av det som skjedde på Utøya 22. juli.	☐	☐	☐	☐	☐	27
• Nye D4 Det som hendte var så motbydelig og frastøtende at jeg føler meg dårlig, blir kvalm eller føler avsky når jeg tenker på det.	☐	☐	☐	☐	☐	28
• Nye D2 Jeg tenker at verden er utrygg, og at jeg ikke er godt nok beskyttet.	☐	☐	☐	☐	☐	29
• Nye D3 Jeg har sterke følelser om hevn	☐	☐	☐	☐	☐	30
• Nye D3 Jeg klandrer meg selv for at jeg lot det som skjedde på Utøya 22. juli endre meg så mye.	☐	☐	☐	☐	☐	31
• Nye D4 Jeg føler meg veldig redd.	☐	☐	☐	☐	☐	32
• Nye D2 Jeg tenker negativt om meg selv, for eksempel at jeg er et dårlig menneske, eller et menneske som ikke stoler på andre.	☐	☐	☐	☐	☐	33

Når du blir minnet på det som skjedde

Q38 **Det er vanlig at ulike situasjoner eller ting omkring oss minner oss om fæle ting som har skjedd. I denne sammenhengen kaller vi dette påminnere. Slike påminnere kan være bestemte steder du går, mennesker eller ting du ser, lyder du hører, ting du ser på TV, eller følelser. Fortell meg hva som minner deg om hendelsene på Utøya 22. juli. Beskriv (så mange som de kommer på):**

Noter ______________________________

Depresjon og angst

Q39 Instruksjon, les opp:

Jeg vil nå spørre deg om noen plager man kan ha uten at de nødvendigvis er relatert til det man har opplevd. Angi i hvilken grad du har vært plaget av hvert enkelt problem i løpet av DE SISTE 14 DAGENE. Sett ett kryss for hver linje.

	Ikke plaget<br>1	Litt plaget<br>2	Ganske mye plaget<br>3	Veldig mye plaget<br>4	
• Stadig redd eller engstelig	☐	☐	☐	☐	1
• Nervøsitet, indre uro	☐	☐	☐	☐	2
• Følelse av håpløshet med tanke på fremtiden	☐	☐	☐	☐	3
• Nedtrykt, tungsindig	☐	☐	☐	☐	4
• Mye bekymret eller urolig	☐	☐	☐	☐	5
• Følelse av at alt er et slit	☐	☐	☐	☐	6
• Følelse av å være anspent, oppjaget	☐	☐	☐	☐	7
• Plutselig frykt uten grunn	☐	☐	☐	☐	8
• Tanker om å ta ditt liv	☐	☐	☐	☐	9

DERSOM UNGDOMMEN SVARER POSITIVT PÅ DETTE SPØRSMÅLET, SKAL DET TAS OPP PÅ SLUTTEN AV INTERVJUET.

Redsel

Q40 **Her kommer noen påstander om hvordan man kan føle seg. I hvilken grad stemmer utsagnene med hvordan du har hatt det DE SISTE 3 MÅNEDENE?**

	Stemmer ikke / stemmer nesten aldri<br>1	Ganske godt / stemmer noen ganger<br>2	Stemmer veldig godt / stemmer ofte<br>3	
• Jeg er redd for å sove alene	☐	☐	☐	1
• Jeg er redd for å være alene hjemme	☐	☐	☐	2
• Jeg liker ikke å være borte fra familien min	☐	☐	☐	3

20©11 Synovate

Dissosiasjon

Q41 **Har du opplevd eller følt noe av dette siden det som skjedde på Utøya 22. juli?**

Instruksjon: Det dreier seg ikke om dissosiasjon dersom ungdommen har vært påvirket av narkotika/alkohol/medikamenter. I så fall, svar nei på spørsmålet

	Ja<br>1	Nei<br>2	
• Har du noen ganger sterke følelser eller tanker som ikke virker som om de er dine, eller følelse av at kroppen din ikke tilhører deg, som om du står utenfor deg selv for eksempel som du ser deg selv på en film?	☐	☐	1
• Er det ting som har skjedd eller ting om deg selv som du har blitt fortalt, men som du ikke selv kan huske?	☐	☐	2
• Har du opplevd at tiden har gått uten at du er sikker på hva du har gjort? Eller være et sted uten å huske hvordan du kom dit?	☐	☐	3
• Har du noen gang vært forvirret med hensyn til hvem du er eller følt deg som en annen person, eller flere personer?	☐	☐	4
• Har det hendt at du plutselig har mistet følelsen i kroppen, mistet evnen til å snakke, mistet synet eller hørselen eller plutselig ikke klart å svelge?	☐	☐	5

Sorg

Q42

Instruksjon: Hvis en nær person (svart JA i **q25**) eller noen i fylkeslaget til ungdommen døde i terrorangrepet, (svart JA i **q31**) spør om sorg. (Hvis det er mer enn et tap ber du ungdommen svare i forhold til det tapet som er vanskeligst):

Du har fortalt meg at du mistet .... (si navnet, ikke skriv det) i terrorangrepet. Jeg vil nå stille deg noen spørsmål om hvordan det har vært for deg etter at .... døde.

	Ikke i det hele tatt<br>1	Noe<br>2	Mye<br>3	
• Hvor vanskelig synes du nå det er å godta at __________ er død?	☐	☐	☐	1
• Hvor mye påvirker sorgen din livet ditt nå?	☐	☐	☐	2
• Hvor mye er du plaget av bilder av eller tanker på _________ når hun/han døde, eller av andre tanker omkring dødsfallet som virkelig plager deg?	☐	☐	☐	3
• Er det ting du pleide å gjøre mens ____________ var i live som du ikke lenger er komfortabel med å gjøre, og som du unngår? Som for eksempel å gå steder dit du gikk sammen med henne/ham, eller gjøre ting som dere likte å gjøre sammen? Eller unngå å se på bilder av eller snakke om _____________ ? Hvor mye unngår du disse tingene?	☐	☐	☐	4
• Hvor mye føler du deg avskåret eller fjern fra andre mennesker etter at ___________ døde, også mennesker du pleide å være nær, som familie eller venner?	☐	☐	☐	5
• Lengter du etter den som døde?	☐	☐	☐	6

20©11 Synovate

Begravelser

Q43 **Har du vært i begravelser relatert til terrorangrepene 22. juli 2011?**

Ja .... ☐ 1
Nei .... ☐ 2

HVIS JA

Q44 **Hvor mange?**

Noter antall ☐ 1

Somatiske helseplager

Q45 **Under er en liste over kroppslige plager man noen ganger kan ha. Hvor mye ha du vært plaget av dette i løpet av DE SISTE 2 UKENE?**

	Ikke plaget 1	Litt plaget 2	Ganske mye plaget 3	Veldig mye plaget 4	
• Vondt i magen (mageknip)	☐	☐	☐	☐	1
• Vondt i hodet	☐	☐	☐	☐	2
• Vondt i korsryggen	☐	☐	☐	☐	3
• Vondt i armer eller bein	☐	☐	☐	☐	4
• Følelse av å skulle besvime eller svimmelhet	☐	☐	☐	☐	5
• Hjertet slår for fort (selv når du ikke trener)	☐	☐	☐	☐	6
• Kvalme eller mageproblem (følelse av å ville kaste opp, eller av at magen er i ulage)	☐	☐	☐	☐	7
• Kraftløshet (å føle seg svak) i deler av kroppen	☐	☐	☐	☐	8

Hodepine

Q46 **Nå vil jeg spørre deg litt mer om hodepine.**

Har du hatt tilbakevendende hodepine i løpet av DE SISTE 3 MND, (som ikke skyldes forkjølelse eller annen sykdom)?

Nei .... ☐ 1
Ja .... ☐ 2

20©11 Synovate

Q47

Instruksjon: Hvis ungdommen svarer Ja, les først begge beskrivelsene nedenfor og be ham eller henne angi hvilke(t) alternativ som passer, og symptomhyppighet. Enkelte kan ha begge typer hodepine. Kryss da av for begge alternativer. For noen passer verken beskrivelse 1 eller 2. Kryss da av for alternativ 3.

Jeg leser nå opp en beskrivelse av to typer hodepine, så kan du angi om en, begge eller ingen av alternativene beskriver dine plager.

	Nei	Mindre enn en gang per mnd	1-3 dager per mnd	1-4 dager per uke	Mer enn 4 dager per uke	
• **Alternativ 1:** Min hodepine kommer ofte som anfall, den er middels sterk eller sterk, og kan begynne på den ene siden. Hodepinen er bankende (dunkende) eller pulserende og jeg føler meg ofte kvalm eller må kaste opp. Når jeg har hodepine tåler jeg dårlig sterkt lys eller lyd, og vil helst ikke bevege hodet.	1 ☐	2 ☐	3 ☐	4 ☐	5 ☐	1
• **Alternativ 2:** Min hodepine kommer ofte smygende, den er lett eller middels sterk og sitter ofte i hele hodet (eller nakken eller midt oppå hodet). Den føles som et press eller trykk (som et bånd rundt hodet), eller er verkende. Vanligvis er jeg ikke kvalm eller må kaste opp. Når jeg har denne hodepinen klarer jeg vanligvis å fortsette med for eksempel skolearbeid eller lekselesing og vanlig fysisk aktivitet.	☐	☐	☐	☐	☐	2
• **Alternativ 3:** Verken beskrivelse 1 eller 2 passer med mine hodepineplager.	☐	☐	☐	☐	☐	3

Alkohol/medikamenter

Q48 **Har du drukket alkohol i løpet av de siste 4 ukene?**

Ja .... ☐ 1
Nei .... ☐ 2

HVIS NEI, GÅ TIL **q51**

Q49 **Omtrent hvor mange ganger har du drukket alkohol i løpet av de siste 4 ukene?**

1-2 ganger .... ☐ 1
3-5 ganger .... ☐ 2
6-9 ganger .... ☐ 3
10-19 ganger .... ☐ 4
Oftere .... ☐ 5

Q50 **Omtrent hvor mange ganger har du kjent deg tydelig beruset i løpet av de siste 4 ukene?**

Ingen ganger .... ☐ 1
1-2 ganger .... ☐ 2
3-5 ganger .... ☐ 3
6-9 ganger .... ☐ 4
10 ganger eller mer .... ☐ 5

Q51 **I løpet av de siste 4 ukene, hvor ofte har du brukt følgende medisiner?**

Sett et kryss på hver linje

	Daglig 1	Hver uke, men ikke daglig 2	Sjeldnere enn hver uke 3	Ikke brukt siste 4 uker 4	
• Smertestillende medisiner	☐	☐	☐	☐	1
• Sovemedisin	☐	☐	☐	☐	2
• Beroligende medisin	☐	☐	☐	☐	3
• Medisin mot depresjon	☐	☐	☐	☐	4

Sosial støtte

Q52 **Nå skal jeg lese opp noen påstander om omsorg og støtte. Jeg vil be deg om å vurdere disse påstandene i forhold til din egen situasjon nå.**

	Så mye jeg ønsker 1	Nesten så mye jeg ønsker 2	Noe, men jeg ville ønsket mer 3	Mindre enn jeg ønsker 4	Mye mindre enn jeg ønsker 5	
• Jeg har mennesker rundt meg som bryr seg om hva som skjer med meg	☐	☐	☐	☐	☐	1
• Jeg får kjærlighet og omsorg	☐	☐	☐	☐	☐	2
• Jeg har mulighet til å snakke med noen om arbeid-/skole-/studieproblemene mine.	☐	☐	☐	☐	☐	3
• Jeg har mulighet til å snakke med noen jeg stoler på om mine personlige problemer eller familieproblemer	☐	☐	☐	☐	☐	4
• Jeg blir bedt med ut for å gjøre ting med andre mennesker.	☐	☐	☐	☐	☐	5
• Jeg får nyttige råd om viktige ting i livet.	☐	☐	☐	☐	☐	6
• Jeg får hjelp når jeg ligger syk til sengs.	☐	☐	☐	☐	☐	7

Skole

SPØRSMÅLENE **q53** TIL **q58** BESVARES BARE AV DE SOM GÅR PÅ SKOLE ELLER STUDERER

Q53 **Startet du på en ny skole/studiested denne høsten?**

Ja ☐ 1
Nei ☐ 2

Q54 **Kontaktet skolen deg for å tilrettelegge din skole/studiestart?**

Ja ☐ 1
Nei ☐ 2

Q55 **Har skolen gjort noe spesielt for å ivareta din læringssituasjon, fordi du var på Utøya?**

Ja ☐ 1
Nei ☐ 2

Q56 **Opplever du at dine skoleprestasjoner har endret seg i forhold til forrige skoleår?**

Dårligere ☐ 1
Uendret ☐ 2
Bedre ☐ 3

Q57 **Har din trivsel på skolen endret seg i forhold til forrige skoleår?**

Dårligere ☐ 1
Uendret ☐ 2
Bedre ☐ 3

Q58 **Har du en som er ansatt på skolen/studiestedet som du kan snakke med dersom du trenger det?**

Ja ☐ 1
Nei ☐ 2

20©11 Synovate

Avhørserfaringer

Q59 **Nå stiller jeg deg noen spørsmål om politiavhør i forbindelse med Utøya. Har du vært i avhør etter hendelsen på Utøya?**

	Ja 1	Nei 2	
• Politiavhør	☐	☐	1
• Dommeravhør	☐	☐	2

HVIS NEI PÅ BEGGE, HOPP OVER RESTEN AV SPØRSMÅLENE OM AVHØRSERFARINGER, OG GÅ TIL **q63**

Q60 **Hvor ble avhøret gjennomført?**

Kryss av det som er korrekt

- På Barnehus ☐ 1
- Hos politiet ☐ 2
- Hjemme ☐ 3
- Annet sted ☐ 4

Q61 **Hvor lenge varte selve avhøret?**

- Under 1 time ☐ 1
- f.o.m. 1 time til 2 timer ☐ 2
- f.o.m. 2 timer til 3 timer ☐ 3
- 3 timer eller mer ☐ 4

Instruks til intervjuer: Les opp:

Fortell hvordan du opplevde å bli avhørt av politiet.

Pass på at diktafonen står på

La informanten fortelle mest mulig fritt

Hjelp informanten videre ved å si: Hva opplevde du?

Hjelp informanten til å utdype ved å si: Fortell meg om det. Eller gjenta det siste de sa.

Q62 (Instruksjon: les høyt)

Nå skal jeg stille deg noen mer detaljerte spørsmål om avhøret.

I hvilken grad...

	Ikke i det hele tatt 1	I liten grad 2	Til dels 3	I stor grad 4	I meget stor grad 5	
• Opplevde du avhøret som nyttig for deg?	☐	☐	☐	☐	☐	1
• Opplevde du avhøret belastende?	☐	☐	☐	☐	☐	2
• Opplevde du at du ble lyttet til og forstått da du fortalte om dine opplevelser fra Utøya i avhør?	☐	☐	☐	☐	☐	3
• Fikk du sterke følelsesmessige reaksjoner som var vanskelig å håndtere under avhøret?	☐	☐	☐	☐	☐	4
• Fikk du sterke følelsesmessige reaksjoner som var vanskelig å håndtere i dagene etter avhøret?	☐	☐	☐	☐	☐	5

Spørsmålene **q63** til **q107** fylles ut av informanten med assistanse av intervjuer.

Husk å gjøre vurdering av udekket hjelpebehov og om neste kontakt før du avslutter!

Hjelpetiltak og besøk på Utøya

Q63 **Var du med på en av tilbakereisene til Utøya**

Ja	☐ 1
Nei	☐ 2

HVIS JA

Q64 **I hvilken grad opplevde du tilbakereisen som nyttig?**

Ikke i det hele tatt	☐ 1
I liten grad	☐ 2
I noen grad	☐ 3
I stor grad	☐ 4
I meget stor grad	☐ 5

Q65 **I hvilken grad opplevde du tilbakereisen som en belastning?**

Ikke i det hele tatt	☐ 1
I liten grad	☐ 2
I noen grad	☐ 3
I stor grad	☐ 4
I meget stor grad	☐ 5

Q66 **Har du hatt kontakt med følgende hjelpeinstanser etter hendelsen?**

	Ja (1)	Nei (2)	
• Tok noen fra kriseteamet eller andre ansatte fra kommunen/bydelen kontakt med deg rett etter hendelsen?	☐	☐	1
• Har du hatt en kontaktperson i kommunen/bydelen?	☐	☐	2

Q67

Q67.A **Etter hendelsene på Utøya 22. juli, har du fått hjelp fra ...?**

Q67.B **Hvis ja, ca hvor mange ganger?**

Q67.C **I hvilken grad har denne hjelpen vært til nytte for deg?**

	Ja (1)	Nei (2)	Noter antall	Ikke i det hele tatt (1)	I noen grad (2)	I stor grad (3)	
• **Fastlegen din**	☐	☐		☐	☐	☐	1
• **Andre hjelpepersoner i kommunen, for eksempel helsesøster, PPT, kommunepsykolog eller andre**	☐	☐		☐	☐	☐	2
• **Psykolog eller psykiater i spesialisthelsetjenesten, for eksempel BUP, DPS eller andre**	☐	☐		☐	☐	☐	3
• **Sjelesørger (prest, imam og lignende)**	☐	☐		☐	☐	☐	4

Q68 **Har noen ansatte eller tillitsvalgte fra AUF vært i kontakt med deg som del av oppfølgingen etter hendelsene på Utøya 22. juli?**

Ja	☐ 1
Nei	☐ 2

HVIS JA

Q69 **I hvilken grad har du opplevd kontakten med AUF som støttende?**

Ikke i det hele tatt	☐ 1
I noen grad	☐ 2
I stor grad	☐ 3

20©11 Synovate

Q70 **Hvor nyttig har kontakten med AUF vært for deg etter terrorangrepet 22. juli?**

Ikke i det hele tatt .... ☐ 1
I noen grad .... ☐ 2
I stor grad .... ☐ 3

Q71 **Dersom du var innlagt i sykehus:**

	Ikke i det hele tatt	I liten grad	Til dels	I høy grad	I meget høy grad	
	1	2	3	4	5	
• I hvilken grad er du tilfreds med mottaket og behandlingen du fikk på sykehuset du var innlagt på?	☐	☐	☐	☐	☐	1
• I hvilken grad er du tilfreds med måten du ble tatt hånd om av legene på sykehuset?	☐	☐	☐	☐	☐	2
• I hvilken grad er du tilfreds med måten du ble tatt hånd om av sykepleierne på sykehuset?	☐	☐	☐	☐	☐	3
• I hvilken grad er du tilfreds med informasjonen du fikk om tilstanden din?	☐	☐	☐	☐	☐	4
• I hvilken grad opplevde du eventuell mottatt psykologisk hjelp på sykehuset som nyttig?	☐	☐	☐	☐	☐	5
• I hvilken grad fikk du nok forklaring på hvilken oppfølging du skulle få etter utskrivning?	☐	☐	☐	☐	☐	6

Hvordan har du det nå

Q72 **Hvor fornøyd er du med livet sånn i alminnelighet på en skala fra 1 til 10, der 1 er svært misfornøyd og 10 er svært fornøyd.?**

	Svært misfor-nøyd									Svært fornøyd	
	01	02	03	04	05	06	07	08	09	10	
... . .	☐	☐	☐	☐	☐	☐	☐	☐	☐	☐	1

Q73 **Fungering**

	Ikke i det hele tatt	Litt av tiden	Noe av tiden	Mye av tiden	Svært mye av tiden	
	0	1	2	3	4	
Synes du det er vanskelig å få gjort det du skal?	☐	☐	☐	☐	☐	1
Er det vanskeligere å komme overens med og/eller være sammen med familie/ venner?	☐	☐	☐	☐	☐	2
Er du mye mindre interessert i, eller får ikke gjort, de tingene du pleide å gjøre før 22. juli?	☐	☐	☐	☐	☐	3

Holdninger, tillit og trygghet

Q74 **Nå skal jeg stille deg noen spørsmål om sikkerhet, overvåking og åpenhet i samfunnet. Hvor enig eller uenig er du i følgende påstander:**

Helt enig – delvis enig – verken eller – delvis uenig – helt uenig

	Helt enig 1	Delvis enig 2	Verken eller 3	Delvis uenig 4	Helt uenig 5	
• Norsk politi bør begynne å bære våpen	☐	☐	☐	☐	☐	1
• I Norge kan man møte sentrale politikere på gaten uten synlig sikkerhet. Slik bør det fortsatt være.	☐	☐	☐	☐	☐	2
• Sikkerhetstiltakene i Norge bør styrkes, selv om det kan gi ulemper for meg selv.	☐	☐	☐	☐	☐	3

Q75 **Hvor bekymret er du for nye terrorangrep i Norge i nær framtid? Oppgi svaret på en skala fra 0 til 10 der 0 er ikke bekymret i det hele tatt og 10 er svært bekymret?**

	Ikke bekymret i det hele tatt 00	01	02	03	04	05	06	07	08	09	Svært bekymret 10	
... .	☐	☐	☐	☐	☐	☐	☐	☐	☐	☐	☐	1

Q76 **På en skala fra 0 til 10 der 0 står for at du ikke har noen tillit i det hele tatt, mens 10 står for at du har full tillit.**

	Ingen tillit i det hele tatt 00	01	02	03	04	05	06	07	08	09	Full tillit 10	
• Hvor stor tillit har du til rettsvesenet?	☐	☐	☐	☐	☐	☐	☐	☐	☐	☐	☐	1
• Hvor stor tillit har du til politiet?	☐	☐	☐	☐	☐	☐	☐	☐	☐	☐	☐	2

Bruk av sosiale medier

Q77 **Har du brukt sosiale medier (for eksempel Facebook, Twitter, Blogg etc.) i tilknytning til terroren 22. juli?**

Ja .... ☐ 1
Nei .... ☐ 2

HVIS JA

Q78 **Hva har du brukt sosiale medier til?**

Kryss av på de svaralternativene som passer best

Lest om andres tanker eller meninger om hendelsene .... ☐ 1,
Delt egne tanker eller meninger om hendelsene .... ☐ 2,
Søkt støtte fra andre .... ☐ 3,
Gitt støtte til andre .... ☐ 4.

20©11 Synovate

Demografi:

Q79 Kjønn

Kvinne/jente .... ☐ 1
Mann/gutt .... ☐ 2

Q80 Antall søsken (inklusive halvsøsken og stesøsken)

Noter antall ☐ 1

Q81 Antall foreldre som er i live (biologiske og adoptivforeldre)

0 .... ☐ 0
1 .... ☐ 1
2 .... ☐ 2

Q82 Er foreldrene dine skilt?

Ja .... ☐ 1
Nei .... ☐ 2

Q83 Antall steforeldre som du bor med

0 .... ☐ 0
1 .... ☐ 1
2 .... ☐ 2

Q84 Bor du sammen med noen?

Nei .... ☐ 1
Ja .... ☐ 2

Q85 Hvis ja, hvem?

Flere svar mulig

Ektefelle/samboer .... ☐ 1,
En eller begge foreldre .... ☐ 2,
Andre voksne .... ☐ 3,
Ett eller flere barn (under 18 år) .... ☐ 4.

Q86 Har du flyttet til annen kommune etter 22. juli?

Ja .... ☐ 1
Nei .... ☐ 2

Q87 I hvilket land er du født : Norge?

Ja .... ☐ 1
Nei .... ☐ 2

HVIS FØDT I ANNET LAND

Q88 Hvilket land ble du født i?

Noter ______________________

____

Q89 I hvilket land er moren din født: Norge?

Ja .... ☐ 1
Nei .... ☐ 2

HVIS MOREN DIN BLE FØDT I ANNET LAND

Q90 Hvilket land ble hun født i?

Noter ______________________

____

Q91 I hvilket land er faren din født: Norge?

Ja .... ☐ 1
Nei .... ☐ 2

HVIS FAREN DIN BLE FØDT I ANNET LAND

Q92 Hvilket land ble han født i?

Noter ______________________

____

Utdanning/arbeid/økonomi

Q93 **Hva er din høyeste fullførte utdanning/skolegang?**

- Universitet/høyskole mer enn 4 år ☐ 1
- Universitet /høyskole inntil 4 år ☐ 2
- Studieforberedende opplæring / allmennfaglig studieretning på videregående skole ☐ 3
- Yrkesfaglig opplæring / yrkesfaglig studieretning på videregående skole ☐ 4
- Grunnskole ☐ 5
- Ingen fullført utdannelse ☐ 6

Q94 **Er du i arbeid (hel- eller deltid, minst 50%)?**

- Ja ☐ 1
- Nei ☐ 2

Q95 **Er du student eller skoleelev (hel- eller deltid, minst 50%)?**

- Ja ☐ 1
- Nei ☐ 2

HVIS JA PÅ SPM. **q94**

Q96 **Har du vært borte fra jobb på grunn av fysiske eller psykiske plager i løpet av de siste 3 månedene?**

- Nei ☐ 1
- Ja ☐ 2

HVIS JA PÅ SPM. **q95**

Q97 **Har du vært borte fra skole på grunn av fysiske eller psykiske plager i løpet av de siste 3 månedene?**

- Nei ☐ 1
- Ja ☐ 2

HVIS JA PÅ SPM. **q96** ELLER **q97**

Q98 **Omtrent hvor mange dager med sykefravær har du hatt til sammen i denne perioden ?**

- 5 dager eller mindre ☐ 1
- 6-10 dager ☐ 2
- 11-20 dager ☐ 3
- Mer enn 20 dager ☐ 4

TIL DEM SOM BOR MED FORELDRE

Q99 **Hvor god råd synes du at familien din har i forhold til folk flest?**

- Mye bedre råd ☐ 1
- Noe bedre råd ☐ 2
- Omtrent som folk flest ☐ 3
- Noe dårligere råd ☐ 4
- Mye dårligere råd ☐ 5

TIL DEM SOM IKKE BOR MED FORELDRE

Q100 **Hvor god råd synes du at du har i forhold til folk flest?**

- Mye bedre råd ☐ 1
- Noe bedre råd ☐ 2
- Omtrent som folk flest ☐ 3
- Noe dårligere råd ☐ 4
- Mye dårligere råd ☐ 5

Tilhørighet i AUF:

Q101 **Var du medlem i AUF 22. juli 2011?**

- Ja ☐ 1
- Nei ☐ 2

HVIS JA

Q102 **Hvor lenge har du vært medlem?**

Antall år ☐ 1

Q103 **Var du tillitsvalgt?**

- Ja ☐ 1
- Nei ☐ 2

20©11 Synovate

Religion:

Q104 **Er du medlem i statskirken eller annet kristent trossamfunn?**

Ja .... ☐ 1
Nei .... ☐ 2

Q105 **Er du medlem av islamsk trossamfunn?**

Ja .... ☐ 1
Nei .... ☐ 2

Q106 **Er du medlem av noen andre trossamfunn?**

Ja .... ☐ 1
Nei .... ☐ 2

Q107 **I hvilken grad er religion viktig for deg personlig?**

I svært stor grad .... ☐ 1
I liten grad .... ☐ 2
Ikke i det hele tatt .... ☐ 3

Når du har fylt inn spørsmålene ovenfor, vil intervjuer avslutte med å diskutere hjelpebehov med deg.

20©11 Synovate

Udekket hjelpebehov?

Spørsmålene nedenfor fylles ut av intervjuer i samråd med deg.

Q108

Hvis informanten svarte litt eller mer plaget på spørsmål om «Tanker om å ta ditt liv» (**svar fra Q39.A.9**) foretas en suicidalitetsvurdering hvor blant annet følgende momenter bør dekkes:

	Ja 1	Nei 2	
Har du noen gang skadet deg selv?	☐	☐	1
Har du skadet deg selv i løpet av de siste 14 dagene?	☐	☐	2
Har du konkrete tanker om å ta ditt eget liv?	☐	☐	3
Har du planer om å ta ditt eget liv?	☐	☐	4

FORETA EN KLINISK VURDERING AV EVENTUELT AKUTT HJELPEBEHOV.

FOR ALLE

Q109

Får du dekket de behovene du mener du har for hjelp etter det som skjedde på Utøya 22.juli (for eksempel fra psykolog, lege, sykepleier, sosial arbeider, eller andre profesjonelle)?

Ja ☐ 1
Nei ☐ 2

Q110

Til intervjuer: Er det kommet fram noe i intervjuet som tilsier at du bør drøfte hjelpebehov med informanten?

Ja ☐ 1
Nei ☐ 2

Q111

Hvis ja, hva ble konklusjonen på drøftelsen? (beskriv)

Noter ____________________

____

Noter ____________________

____

Noter ____________________

____

20©11 Synovate

Q112 **Hvordan er dette fulgt opp av intervjueren?**

Dette kan for eksempel være at kontaktperson i kommunen er kontaktet og henviser informanten til psykisk helsevern, eller at man har kommet fram til at det ikke er behov for ytterligere hjelp.

Noter ______________________________

____

Noter ______________________________

____

Noter ______________________________

____

Q113 **Ny kontakt**

Når intervjuet avsluttes, spør intervjueren om det er i orden at vi kontakter igjen om ca 6 måneder for nytt intervju.

Ja .... ☐ 1
Nei .... ☐ 2

Avslutning av intervjuet

(Til intervjueren: Gi ungdommen god tid til å avslutte intervjuet eller kommentere. Kanskje er det ting de ønsker du skulle ha spurt om eller annet de ønsker å formidle tilbake til oss. Dersom ungdommen trenger tid til å avslutte, vis respekt for det. Hjelp til med eventuelle emosjonelle reaksjoner som de trenger hjelp til. Husk å takke for at de har deltatt og minn dem på at all kontaktinformasjon står i informasjonsskrivet.)

20©11 Synovate
