# Supplementary material for: Health service utilization after terrorism: a longitudinal study of survivors of the 2011 Utøya attack in Norway
Source: BMC Health Serv Res. 2015 Apr 15;15:158. doi: 10.1186/s12913-015-0811-6 (PMC4457986; doi:10.1186/s12913-015-0811-6)
Supplement: Additional file 2: — Interview questionnaire at T2 (in Norwegian). [file 12913_2015_811_MOESM2_ESM.pdf]

**Prosjekt**	**1076481**
**Skjemanummer**	

Informasjon til intervjuer

Husk at samtykkeerklæringen skal undertegnes før intervjuet starter. Vi er opptatt av hvordan du har det, og det kommer vi til å snakke mye om i intervjuet.

Husk å fylle ut kontaktinformasjon samtidig med samtykkeerklæringen.

STILL ALLE SPØRSMÅL – selv om du synes du har spurt om dette før. Hvis informanten ikke forstår spørsmålet, gjenta det. Deretter kan du prøve å forklare hva det betyr, men unngå å utdype med din egen tolkning av meningsinnholdet i spørsmålet. Alt som står i grå bokser er info til intervjuer. Ikke les dette høyt.

Intervjumanualen består av 4 deler:

Del I består av flere åpne spørsmål eller narrativ, hvor vi ønsker fyldige beskrivelser fra informanten. Hvis informanten har vanskeligheter med å svare eller svarer kort på spørsmålene, hjelp informanten videre ved å spørre oppfølgingsspørsmål i tråd med instruksjonene under hvert enkelt spørsmål. «Åpningsspørsmålet» er ment som et innledende spørsmål for å møte informantene der de er nå. Spørsmålet om ¨Rettssaken¨ går til alle informantene. Spørsmålene om «Skole», «Sosiale medier» og «Identitet og tilhørighet» skal bare besvares av et delutvalg. Følg instruksjonene underveis. Bruk ca. 5-10 minutter per tema, ca. 30 minutter til sammen på Del I.

Del II består av strukturerte spørsmål som alle skal svare på, og som intervjuer leser opp.

Del III skal informanten fylle ut selv med assistanse av intervjuer. Her kommer noen tema på nytt (noen av temaene fra Del I). Alle skal svare på disse og følge instruksjonene underveis.

Del IV er en avslutning av intervjuet hvor intervjuer skal vurdere hvorvidt det er et udekket hjelpebehov som må følges opp av intervjuer.

Introduksjon

I fjor snakket vi med mange av dere som var på Utøya 22. juli, og vi snakket med mange av foreldrene deres. Sist vi snakket sammen var vi opptatt av hva dere opplevde, og hvordan dere hadde det da. Vi er fremdeles opptatt av hvordan dere har det, og det kommer vi til å snakke mye om i intervjuet. Grunnen til dette er at det vil kunne gi oss viktig kunnskap om hvilke belastninger slike hendelser har i et lengre tidsperspektiv.

Det du forteller meg vil ikke bli fortalt videre til dine foreldre, på samme måte som du ikke får vite hva dine foreldre forteller oss. Hvis du sier noe som gjør at jeg blir bekymret for deg, må vi drøfte hva vi kan gjøre for å hjelpe deg videre. Dersom du er under 18 år kan det hende jeg må snakke med foreldrene dine om dette, men da vil jeg si ifra til deg først.

Si ifra til meg hvis det er noe du ikke forstår, eller hvis det er noe du synes det er vanskelig å svare på. Hvis du vil ha en pause så er det helt i orden. Dersom det er spørsmål du ikke ønsker å svare på, kan du si fra underveis. Du trenger ikke å forklare hvorfor du ikke ønsker å svare på spørsmål.

Jeg har med diktafon, slik at vi etter intervjuet kan skrive ned det du forteller oss. Er det i orden for deg?

SLÅ PÅ DIKTAFONEN

20©12 Ipsos MMI

Instruksjon til intervjuer:

Skjemaet avleses maskinelt. Pass derfor på at du setter kryss innenfor den avmerkede boksen. Skriv tydelige tall der du blir bedt om å skrive tall.

ID **NB! Skriv inn id.nr. og les det høyt opp på begynnelsen av intervjuet.**

ID .... [| | |] 1

ID **Dagens dato**

Eks. 1. september 2012 noteres slik: 010912

Noter .... [| | | | |] 1

Del I Åpne spørsmål

Åpningsspørsmål

Nå er det gått over ett år siden hendelsene 22. juli i fjor, og det er mye som har skjedd i løpet av denne tiden. Fortell meg hvordan dette siste året har vært for deg.

Rettssaken etter 22. juli 2011

Rettssaken etter 22.juli 2011 er over etter å ha vart i 10 uker, og dommen er avsagt. Tenk deg tilbake til tiden da rettssaken pågikk. Fortell meg hvordan du opplevde rettssaken.

(Hvis du ikke fulgte med i rettssaken, ønsker jeg å få vite bakgrunnen for dette.)

Instruksjon til intervjuer:

Vi ønsker både å få frem om- og hvordan ungdommen har engasjert seg i rettssaken, og hvordan den følelsesmessig har virket inn. Og om det er deler av rettssaken som har gjort spesielle inntrykk: eksempelvis gjerningsmannens forklaring, obduksjonsrapportene, vitneforklaringer.

Skole

S1 **Gikk du i 2. eller 3. klasse på videregående skole hele eller deler av skoleåret 2011/12?**

Ja .... ☐ 1

Nei .... **(⇒ S2)** ☐ 2

Fortell hvordan forrige skoleår var for deg, på godt og vondt. Jeg tenker spesielt med bakgrunn i dine opplevelser fra 22. juli 2011.

NØKKELTEMA:
Faglig fungering, trivsel, sosial/emosjonell støtte og faglige tilrettelegginger på skolen.

20©12 Ipsos MMI

Sosiale medier

S2 **Er du født i 1992 eller tidligere, og har du brukt sosiale medier i tilknytning til hendelsene 22. juli 2011 og tiden etter?**

Ja .... ☐ 1
Nei .... **(⇒ S3)** ☐ 2

Instruksjon til intervjuer:

Deltageren må tilfredsstille begge kriterier for å gå videre til intervju om bruk av sosiale medier.

I de følgende spørsmålene er jeg interessert i all form for kommunikasjon du har hatt gjennom sosiale medier som kan tilknyttes det du opplevde.

Fortell meg om sist gang du brukte sosiale medier i tilknytning til det du opplevde 22. juli 2011 og tiden etter.

NØKKELTEMA:
Hva brukte du sosiale medier til? Hvem kommuniserte du med? Hva kommuniserte du? Hvordan opplevde du det? Hva slags sosiale medier brukte du?

Kan du gi meg noen eksempler på positive erfaringer med å bruke sosiale medier i tilknytning til det du opplevde 22. juli 2011 og tiden etter?

NØKKELTEMA
Hva var det du opplevde som positivt? Hvem kommuniserte du med?

Kan du gi meg noen eksempler på negative erfaringer med å bruke sosiale medier i tilknytning til det du opplevde 22. juli 2011 og tiden etter?

NØKKELTEMA:
Hva var det du opplevde som negativt? Hvem kommuniserte du med?

Tilhørighet

Nå kommer et spørsmål til de som var på Utøya som har minoritetsbakgrunn.

S3 **Er du selv, din mor og/eller din far født i et øst-europeisk, asiatisk, afrikansk eller sør-amerikansk land?**

Ja .... ☐ 1
Nei .... **(⇒ Q1)** ☐ 2

Har din opplevelse av tilhørighet til det norske samfunnet endret seg etter 22. juli 2011, og i så fall på hvilken måte?

NØKKELTEMA:
Eksklusjons-/inklusjonsmekanismer i det norske samfunnet, hvordan oppleves det å tilhøre en etnisk minoritetsgruppe i Norge etter 22. juli 2011.

20©12 Ipsos MMI

Del II Lukkede spørsmål

Posttraumatiske stressreaksjoner

Man kan få ulike reaksjoner på dramatiske hendelser. Nå kommer jeg til å lese opp noen påstander om mulige reaksjoner etter terrorangrepet på Utøya 22. juli 2011. For hver påstand vil jeg spørre hvor ofte du har opplevd å ha det slik DEN SISTE MÅNEDEN.
Her er et skjema som er til hjelp når du skal svare på hvor ofte du har opplevd problemet den siste måneden.

Instruksjon til intervjuer:

Ta frem frekvensarket og legg på bordet mellom dere. Gå gjennom de ulike alternativene før dere starter på listen.

Q1 **Hvor mye av tiden DEN SISTE MÅNEDEN**

	Aldri 1	Sjelden 2	Noen ganger 3	Ofte 4	Nesten alltid 5	
• Jeg er på vakt for fare eller ting jeg er redd for.	☐	☐	☐	☐	☐	1
• Når noe minner meg om det som skjedde på Utøya 22. juli 2011, blir jeg veldig ute av meg, redd eller trist.	☐	☐	☐	☐	☐	2
• Jeg får skremmende tanker, ser for meg bilder eller hører lyder fra det som skjedde på Utøya 22. juli 2011, selv om jeg ikke vil det.	☐	☐	☐	☐	☐	3
• Jeg føler meg sur, sint eller rasende.	☐	☐	☐	☐	☐	4
• Jeg drømmer om det som skjedde på Utøya 22. juli 2011 eller har mareritt om andre ting.	☐	☐	☐	☐	☐	5
• Jeg føler at jeg er tilbake til da terroren skjedde på Utøya 22. juli 2011, og opplever det om igjen.	☐	☐	☐	☐	☐	6
• Jeg har lyst til å være alene og ikke sammen med venner.	☐	☐	☐	☐	☐	7
• Jeg føler meg alene inni meg og føler ikke nærhet til andre mennesker.	☐	☐	☐	☐	☐	8
• Jeg forsøker å ikke snakke om det som skjedde på Utøya 22. juli 2011, tenke på det eller ha følelser rundt det.	☐	☐	☐	☐	☐	9
• Jeg har problemer med å føle glede eller kjærlighet.	☐	☐	☐	☐	☐	10
• Jeg har problemer med å føle tristhet eller sinne.	☐	☐	☐	☐	☐	11
• Jeg blir lett urolig eller skvetter lett, for eksempel når jeg hører høye lyder eller når noe overrasker meg.	☐	☐	☐	☐	☐	12
• Jeg har problemer med å få sove eller jeg våkner ofte om natten.	☐	☐	☐	☐	☐	13
• Jeg bebreider meg selv for noe av det som skjedde på Utøya 22. juli 2011	☐	☐	☐	☐	☐	14
• Jeg har problemer med å huske viktige ting fra det som skjedde på Utøya 22.juli.	☐	☐	☐	☐	☐	15
• Jeg har problemer med å konsentrere meg eller være oppmerksom.	☐	☐	☐	☐	☐	16
• Jeg forsøker å holde meg unna folk, steder eller ting som minner meg om det som skjedde på Utøya 22.juli.	☐	☐	☐	☐	☐	17
• Når noe minner meg om det som skjedde på Utøya 22. juli 2011, får jeg sterke følelser i kroppen, for eksempel hjertebank, hodepine eller mageknip.	☐	☐	☐	☐	☐	18
• Jeg tror ikke at jeg kommer til å leve lenge.	☐	☐	☐	☐	☐	19
• Jeg krangler eller slåss mye	☐	☐	☐	☐	☐	20
• Jeg er negativ eller pessimistisk når jeg tenker på fremtiden	☐	☐	☐	☐	☐	21

Hvor mye av tiden DEN SISTE MÅNEDEN

	Aldri	Sjelden	Noen ganger	Ofte	Nesten alltid	
• Jeg er redd for at noe lignende som det som skjedde på Utøya 22. juli 2011 skal skje igjen.	☐	☐	☐	☐	☐	22
• Jeg er sint på noen andre for at de ikke gjorde mer for å forhindre det som skjedde på Utøya 22. juli 2011, eller for at de ikke har gjort nok etterpå (gjelder ikke sinne mot gjerningsmannen).	☐	☐	☐	☐	☐	23
• Jeg tenker at andre vonde ting vil skje med meg i fremtiden.	☐	☐	☐	☐	☐	24
• Jeg gjør ting som skader eller kan skade meg selv eller andre. (For eksempel å kjøre for fort, bruke narkotika, drikke veldig mye, skade meg selv eller gjøre andre farlige ting).	☐	☐	☐	☐	☐	25
• Jeg klandrer meg selv for at jeg ikke er blitt fortere bra.	☐	☐	☐	☐	☐	26
• Jeg skammer meg over noe av det som skjedde på Utøya 22. juli 2011.	☐	☐	☐	☐	☐	27
• Det som hendte var så motbydelig og frastøtende at jeg føler meg dårlig, blir kvalm eller føler avsky når jeg tenker på det.	☐	☐	☐	☐	☐	28
• Jeg tenker at verden er utrygg, og at jeg ikke er godt nok beskyttet.	☐	☐	☐	☐	☐	29
• Jeg har sterke følelser om hevn	☐	☐	☐	☐	☐	30
• Jeg klandrer meg selv for at jeg lot det som skjedde på Utøya 22. juli 2011 endre meg så mye.	☐	☐	☐	☐	☐	31
• Jeg føler meg veldig redd.	☐	☐	☐	☐	☐	32
• Jeg tenker negativt om meg selv, for eksempel at jeg er et dårlig menneske, eller et menneske som ikke stoler på andre.	☐	☐	☐	☐	☐	33

20©12 Ipsos MMI

Om å bli minnet på hendelsen

Q2 **Det er vanlig at ulike situasjoner, eller ting omkring oss, kan minne oss om fæle ting som har skjedd. Noen ganger kan dette vekke vonde følelser og/eller kroppslige reaksjoner som kan ligne på (minne om) det du kjente under terrorangrepet. Dette kaller vi påminnere. Slike påminnere kan være bestemte steder du går, mennesker eller ting du ser, lyder du hører, ting du ser på TV, eller følelser.**

Jeg skal nå lese opp en liste med mulige påminnere. Jeg vil at du skal fortelle meg hvor ofte, i løpet av DEN SISTE MÅNEDEN, disse tingene har minnet deg om det som skjedde på Utøya òg gjort deg oppskaket eller stresset.

Type påminner	1 Aldri	2 Sjelden	3 Noen ganger	4 Ofte	5 Veldig ofte	
• **Lyder** (Skudd, skrik, skarpe smell, fyrverkeri, alarmer, sirener, helikopter, etc.)	1 ☐	2 ☐	3 ☐	4 ☐	5 ☐	1
• **Synsopplevelser** (Bilder, avisoppslag, uniformer, politi, våpen, Facebook profiler/oppdateringer, etc.)	☐	☐	☐	☐	☐	2
• **Lukt** (Kruttlukt, blodlukt, sykehuslukt, parfyme, lukt av skog eller stein, etc.)	☐	☐	☐	☐	☐	3
• **Berøring** (Kontakt med vann, kroppskontakt, etc.)	☐	☐	☐	☐	☐	4
• **Følelser** (Trist, redd, stresset, tomhet, frykt, etc.)	☐	☐	☐	☐	☐	5
• **Kroppslige reaksjoner** (Stramme muskler, hjertebank, pustevansker, etc.)	☐	☐	☐	☐	☐	6
• **Situasjonsbetinget** Løping, menneskemengder, steder, bygninger/telt, AUF, naturomgivelser (som øyer, vann, skog, regn), sommer, datoer, ettårsdagen	☐	☐	☐	☐	☐	7

Q3 **Andre påminnere (beskriv):**

Noter ______________________________

____

Noter ______________________________

____

Noter ______________________________

____

Q4 **Av alle disse påminnerne, hvilken synes du er den verste (den mest plagsomme)?**

Noter ______________________________

____

20©12 Ipsos MMI

Om å oppleve vanskelige følelser

Q5 **Nå kommer noen spørsmål om tanker og opplevelser man kan ha etter å ha opplevd dramatiske hendelser. Har du opplevd noe av dette på noe tidspunkt siden 22. juli 2011?**

Sett ett kryss i hver linje

	Nei (0)	Ja, litt (1)	Ja, mye (2)	
• Har du bekymret deg over hva andre mennesker tenker om deg etter det som skjedde på Utøya 22. juli 2011?	☐	☐	☐	1
• Har du forsøkt å skjule noe som du gjorde, eller ikke gjorde, 22. juli 2011?	☐	☐	☐	2
• Har du opplevd at andre har trukket seg vekk fra deg eller familien din etter det som skjedde på Utøya 22. juli 2011.	☐	☐	☐	3
• Har du opplevd at andre har klandret deg for noe som hendte på Utøya 22. juli 2011?	☐	☐	☐	4
• Har du noen gang klandret deg selv for at du overlevde mens andre ble drept?	☐	☐	☐	5

Depresjon og angst

Q6 **Jeg vil nå spørre deg om noen plager man kan ha uten at de nødvendigvis er relatert til det man har opplevd. Angi i hvilken grad du har vært plaget av hvert enkelt problem i løpet av DE SISTE 14 DAGENE. Sett ett kryss for hver linje.**

	Ikke plaget (1)	Litt plaget (2)	Ganske mye plaget (3)	Veldig mye plaget (4)	
• Stadig redd eller engstelig	☐	☐	☐	☐	1
• Nervøsitet, indre uro	☐	☐	☐	☐	2
• Følelse av håpløshet med tanke på fremtiden	☐	☐	☐	☐	3
• Nedtrykt, tungsindig	☐	☐	☐	☐	4
• Mye bekymret eller urolig	☐	☐	☐	☐	5
• Følelse av at alt er et slit	☐	☐	☐	☐	6
• Følelse av å være anspent, oppjaget	☐	☐	☐	☐	7
• Plutselig frykt uten grunn	☐	☐	☐	☐	8
• Tanker om å ta ditt liv	☐	☐	☐	☐	9

INSTRUKSJON TIL INTERVJUER:

DERSOM UNGDOMMEN SVARER POSITIVT PÅ DET SISTE SPØRSMÅLET, SKAL DET TAS OPP PÅ SLUTTEN AV INTERVJUET.

20©12 Ipsos MMI

Dissosiasjon

Q7 **Har du opplevd eller følt noe av det følgende i løpet av DEN SISTE MÅNEDEN (når du ikke har vært påvirket av rus)?**

	Aldri	Sjelden	Noen ganger	Ofte	Nesten alltid	
• Har du noen ganger sterke følelser eller tanker som virker som om de ikke er dine, eller følelse av at kroppen din ikke tilhører deg, som om du står utenfor deg selv, for eksempel som om du ser deg selv på film?	☐ 0	☐ 1	☐ 2	☐ 3	☐ 4	1
• Er det ting som har skjedd deg som du selv ikke kan huske, men som har blitt fortalt deg av andre?	☐	☐	☐	☐	☐	2
• Har du opplevd at tiden har gått uten at du er sikker på hva du har gjort? Eller at du kan være et sted uten å huske hvordan du kom dit?	☐	☐	☐	☐	☐	3
• Har du noen gang vært forvirret med hensyn til hvem du er eller følt deg som du er en eller flere andre personer?	☐	☐	☐	☐	☐	4
• Har det hendt at du plutselig har mistet følelsen i kroppen, mistet kraften i ben og armer, fått kramper, mistet evnen til å snakke, mistet synet eller hørselen eller plutselig ikke klart å svelge?	☐	☐	☐	☐	☐	5

Skader

Q8 **Ble du fysisk skadet og hadde behov for medisinsk hjelp under terrorangrepet 22. juli 2011?**

Ja .... ☐ 1
Nei .... **(⇒ Q11)** ☐ 2

Q9 **Har du fysiske skader fra terrorangrepet som hemmer deg i det daglige? (for eksempel i aktiviteter/ fritidsaktiviteter/ skolefungering/ daglige rutiner?)**

Ja .... ☐ 1
Nei .... ☐ 2

Q10 **Hvor plaget er du av skaden(e) nå?**

Ikke i det hele tatt .... ☐ 1
I liten grad .... ☐ 2
Noe .... ☐ 3
I stor grad .... ☐ 4
I veldig stor grad .... ☐ 5

20©12 Ipsos MMI

Somatiske helseplager

Q11 **Jeg skal nå lese opp en liste over kroppslige plager man noen ganger kan ha. Hvor mye har du vært plaget av dette i løpet av DE SISTE 2 UKENE?**

	Ikke plaget 1	Litt plaget 2	Ganske mye plaget 3	Veldig mye plaget 4	
• Vondt i magen (mageknip)	☐	☐	☐	☐	1
• Vondt i hodet	☐	☐	☐	☐	2
• Vondt i korsryggen	☐	☐	☐	☐	3
• Vondt i armer eller bein	☐	☐	☐	☐	4
• Følelse av å skulle besvime eller svimmelhet	☐	☐	☐	☐	5
• Hjertet slår for fort (selv når du ikke trener)	☐	☐	☐	☐	6
• Kvalme eller mageproblem (følelse av å ville kaste opp, eller av at magen er i ulage)	☐	☐	☐	☐	7
• Kraftløshet (å føle seg svak) i deler av kroppen	☐	☐	☐	☐	8

Q12 **Nå vil jeg spørre deg litt mer om hodepine. Hadde du tilbakevendende hodepine i løpet av de siste 3 månedene før 22. juli 2011? Da tenker jeg på hodepine som ikke skyldes forkjølelse eller annen sykdom.**

Ja .... ☐ 1
Nei .... ☐ 2

Q13 **Har du hatt tilbakevendende hodepine i løpet av DE SISTE 3 MND?Da tenker jeg på hodepine som ikke skyldes forkjølelse eller annen sykdom.**

Ja .... ☐ 1
Nei .... **(⇒ Q15)** ☐ 2

Q14 **Jeg leser nå opp to beskrivelser av hodepineplager. Du forteller meg om en, begge eller ingen av beskrivelsene passer for hodepineplager du har hatt i løpet av de 3 SISTE MÅNEDENE.**

	Nei 1	Mindre enn en gang per mnd 2	1-3 dager per mnd 3	1-4 dager per uke 4	Mer enn 4 dager per uke 5	
• **A. Beskrivelse** Hodepinen kommer ofte som anfall, den er middels sterk eller sterk, og kan begynne på den ene siden. Hodepinen er bankende (dunkende) eller pulserende og jeg føler meg ofte kvalm eller må kaste opp. Når jeg har hodepine tåler jeg dårlig sterkt lys eller lyd, og vil helst ikke bevege hodet.	☐	☐	☐	☐	☐	1
• **B. Beskrivelse** Hodepinen kommer ofte smygende, den er lett eller middels sterk og sitter ofte i hele hodet (eller nakken eller midt oppå hodet). Den føles som et press eller trykk (som et bånd rundt hodet), eller er verkende. Vanligvis er jeg ikke kvalm eller må kaste opp. Når jeg har denne hodepinen klarer jeg vanligvis å fortsette med for eksempel skolearbeid eller lekselesing og vanlig fysisk aktivitet.	☐	☐	☐	☐	☐	2
• **C.** Verken beskrivelse A eller B passer med mine hodepineplager.	☐	☐	☐	☐	☐	3

20©12 Ipsos MMI

Sorg

Q15 **Mistet du en nær person (nær venn, kjæreste, familiemedlem) i terrorangrepet?**

- Ja, nær venn ☐ 1,
- Ja, kjæreste ☐ 2,
- Ja, familiemedlem ☐ 3.

Q16 Jeg vil nå stille deg noen spørsmål om hvordan det har vært for deg etter at han/hun døde.

	Ikke i det hele tatt<br>1	Noe<br>2	Mye<br>3	
• Hvor vanskelig synes du nå det er å godta at han/hun er død?	☐	☐	☐	1
• Hvor mye påvirker sorgen livet ditt nå?	☐	☐	☐	2
• Hvor mye er du plaget av bilder av eller tanker på (den som døde) når hun/han døde, eller av andre tanker omkring dødsfallet som virkelig plager deg?	☐	☐	☐	3
• Er det ting du pleide å gjøre mens han/hun var i live som du ikke lenger er komfortabel med å gjøre, og som du unngår? Som for eksempel å gå steder dit du gikk sammen med henne/ham, eller gjøre ting som dere likte å gjøre sammen? Eller unngå å se på bilder av eller snakke om han/hun ? Hvor mye unngår du disse tingene?	☐	☐	☐	4
• Hvor mye føler du deg avskåret eller fjern fra andre mennesker etter at han/hun døde, også mennesker du pleide å være nær, som familie eller venner?	☐	☐	☐	5

Alkohol og medikamenter

Q17 **Har du drukket alkohol i løpet av de siste 4 ukene?**

- Ja ☐ 1
- Nei **(⇒ Q21)** ☐ 2

Q18 **Omtrent hvor mange ganger har du drukket alkohol i løpet av de siste 4 ukene?**

- 1-2 ganger ☐ 1
- 3-5 ganger ☐ 2
- 6-9 ganger ☐ 3
- 10-19 ganger ☐ 4
- Oftere ☐ 5

Q19 **Omtrent hvor mange ganger har du kjent deg tydelig beruset i løpet av de siste 4 ukene?**

- Ingen ganger ☐ 1
- 1-2 ganger ☐ 2
- 3-5 ganger ☐ 3
- 6-9 ganger ☐ 4
- 10 ganger eller mer ☐ 5

20©12 Ipsos MMI

Q20 **I løpet av de siste 4 ukene, hvor ofte har du brukt alkohol for å dempe angst, bli mindre deprimert eller for å få sove?**

- Daglig ☐ 1
- Hver uke, men ikke daglig ☐ 2
- Sjeldnere enn hver uke ☐ 3
- Ikke brukt siste 4 ukene ☐ 4

Q21 **I løpet av de siste 4 ukene, hvor ofte har du brukt følgende medisiner?**

Sett et kryss på hver linje

	Daglig 1	Hver uke, men ikke daglig 2	Sjeldnere enn hver uke 3	Ikke brukt siste 4 uker 4	
• Smertestillende medisiner	☐	☐	☐	☐	1
• Sovemedisin	☐	☐	☐	☐	2
• Beroligende medisin	☐	☐	☐	☐	3
• Medisin mot depresjon	☐	☐	☐	☐	4

Sosial støtte

Q22 **Nå skal jeg lese opp noen påstander om omsorg og støtte. Jeg vil be deg om å vurdere disse påstandene i forhold til din egen situasjon nå.**

	Så mye jeg ønsker 1	Nesten så mye jeg ønsker 2	Noe, men jeg ville ønsket mer 3	Mindre enn jeg ønsker 4	Mye mindre enn jeg ønsker 5	
• Jeg har mennesker rundt meg som bryr seg om hva som skjer med meg	☐	☐	☐	☐	☐	1
• Jeg får kjærlighet og omsorg	☐	☐	☐	☐	☐	2
• Jeg har mulighet til å snakke med noen om arbeid-/skole-/studieproblemene mine.	☐	☐	☐	☐	☐	3
• Jeg har mulighet til å snakke med noen jeg stoler på om mine personlige problemer eller familieproblemer	☐	☐	☐	☐	☐	4
• Jeg blir bedt med ut for å gjøre ting med andre mennesker.	☐	☐	☐	☐	☐	5
• Jeg får nyttige råd om viktige ting i livet.	☐	☐	☐	☐	☐	6
• Jeg får hjelp når jeg ligger syk til sengs.	☐	☐	☐	☐	☐	7

Q23 **Fra tidligere katastrofer vet vi at det finnes ulike barrierer mot å søke sosial støtte. I hvor stor grad føler du at følgende utsagn passer for deg? Jeg lar være å søke hjelp og støtte, eller snakke med andre om hvordan jeg har det, fordi jeg tenker at**

	Ikke i det hele tatt 1	I liten grad 2	Til dels 3	I stor grad 4	I meget stor grad 5	
• De er lei av å høre om det	☐	☐	☐	☐	☐	1
• De har det vanskelig nok selv	☐	☐	☐	☐	☐	2
• De kan syns jeg er for opphengt i det som har skjedd	☐	☐	☐	☐	☐	3
• Jeg ikke skal bruke opp vennene mine	☐	☐	☐	☐	☐	4
• De som ikke var på Utøya, ikke kan forstå meg	☐	☐	☐	☐	☐	5
• Jeg føler meg sviktet av noen som jeg trodde ville støttet meg	☐	☐	☐	☐	☐	6

20©12 Ipsos MMI

Instruksjon til intervjuer:
Her følger en del spørsmål deltageren kan fylle ut selv. Vær tilgjengelig for spørsmål deltageren har. Husk å gjøre vurdering av udekket hjelpebehov før du avslutter intervjuet.

Del III Selvutfylling

Hvordan har du det nå?

Q24 **Hvor fornøyd er du med livet sånn i alminnelighet?**

Sett et kryss på en skala fra 1 til 10, der 1 er svært misfornøyd og 10 er svært fornøyd.

Svært misfornøyd									Svært fornøyd	
01	02	03	04	05	06	07	08	09	10	
☐	☐	☐	☐	☐	☐	☐	☐	☐	☐	1

Q25 **I hvor stor grad er du tilbake i vanlig funksjon på følgende områder?**

Sett ett kryss i hver linje

	Ikke i det hele tatt	I liten grad	I noen grad	I stor grad	Helt tilbake	
	1	2	3	4	5	
• Skole/studier/arbeid	☐	☐	☐	☐	☐	1
• På fritiden	☐	☐	☐	☐	☐	2
• Forhold til venner	☐	☐	☐	☐	☐	3
• Forhold til familien	☐	☐	☐	☐	☐	4
• Husarbeid (plikter hjemme, vaske klær, etc.)	☐	☐	☐	☐	☐	5

Skole

Du skal svare på dette spørsmålet selv om du tidligere i intervjuet har fortalt om ditt forrige skoleår.

Q26 **Gikk du på skole, eller studerte du, hele eller deler av skoleåret 2011/12?**

Ja ☐ 1
Nei **(⇒ Q33)** ☐ 2

Q27 **Fullførte du hele forrige skole/studieår?**

Ja ☐ 1
Nei ☐ 2

Q28 **Opplever du at dine skole/studieprestasjoner har endret seg etter 22. juli 2011?**

Dårligere ☐ 1
Uendret ☐ 2
Bedre ☐ 3

Q29 **Har din trivsel på skolen/studiestedet endret seg etter 22. juli 2011?**

Dårligere ☐ 1
Uendret ☐ 2
Bedre ☐ 3

Q30 **Nå kommer noen spørsmål om dine vurderinger av behov for støtte og tilrettelegginger i forrige skoleår på bakgrunn av dine opplevelser fra 22. juli 2011.**

Sett ett kryss i hver linje

	Ikke i det hele tatt	I liten grad	Til dels	I høy grad	I meget høy grad	
	1	2	3	4	5	
• Hadde du behov for ekstra praktiske tilrettelegginger som f.eks. utsettelse av innleveringer og tilpassede prøver/eksamener?	☐	☐	☐	☐	☐	1
• Hadde du behov for ekstra faglig støtte fra lærere?	☐	☐	☐	☐	☐	2
• Hadde du behov for ekstra sosial- og følelsesmessig støtte på skolen?	☐	☐	☐	☐	☐	3

Q31 **Mottok du ekstra støtte forrige skoleår?**

Ja ☐ 1
Nei (⇒ Q33) ☐ 2

Q32 **Sett under ett, hvor fornøyd er du med støtten/tilretteleggingen skolen ga forrige skoleår?**

Ikke i det hele tatt ☐ 1
I liten grad ☐ 2
Til dels ☐ 3
I høy grad ☐ 4
I meget høy grad ☐ 5

Q33 **Går du på skole eller universitet/høgskole nå?**

Ja ☐ 1
Nei (⇒ Q35) ☐ 2

Q34 **Du vil nå få noen spørsmål om hvordan du synes det er å utføre skolearbeid/studiearbeid. Hvor lett synes du det er å:**

Sett ett kryss i hver linje

	Ikke lett i det hele tatt	Ikke særlig lett	Ganske lett	Lett	Svært lett	
	1	2	3	4	5	
• Gjøre ferdig hjemmeoppgaver innen en tidsfrist?	☐	☐	☐	☐	☐	1
• Gjøre skolearbeid når det er andre interessante ting å gjøre?	☐	☐	☐	☐	☐	2
• Konsentrere deg om skolefag?	☐	☐	☐	☐	☐	3
• Ta notater i timen?	☐	☐	☐	☐	☐	4
• Bruke biblioteket for å få informasjon til klasseoppgaver?	☐	☐	☐	☐	☐	5
• Planlegge skolearbeidet ditt?	☐	☐	☐	☐	☐	6
• Organisere skolearbeidet ditt?	☐	☐	☐	☐	☐	7
• Huske ting som blir sagt i klassen og som står i skolebøkene?	☐	☐	☐	☐	☐	8
• Ordne deg en leseplass der du kan jobbe uten forstyrrelser?	☐	☐	☐	☐	☐	9
• Motivere deg til å gjøre skolearbeid?	☐	☐	☐	☐	☐	10
• Delta i klassediskusjoner?	☐	☐	☐	☐	☐	11

20©12 Ipsos MMI

Politisk aktivitet

Q35 **Er du mer eller mindre politisk aktiv nå sammenlignet med før 22. juli 2011?**

Mindre .... ☐ 1
Uendret .... ☐ 2
Mer .... ☐ 3

Q36 **Har du fått nytt tillitsverv etter 22. juli 2011?**

Ja .... ☐ 1
Nei .... ☐ 2

Q37 **Har du sluttet i tillitsverv etter 22. juli 2011?**

Ja .... ☐ 1
Nei .... ☐ 2

Rettssaken

Du har tidligere i intervjuet fortalt om dine opplevelser knyttet til rettssaken etter 22. juli 2011. Her kommer noen spørsmål om hvor mye du har deltatt i rettssaken.

Q38 **Jeg har fulgt rettsaken i media**

Ja .... ☐ 1
Nei .... ☐ 2

Q39 Hvis ja

Gjennomsnittlig ant. timer pr. dag (under rettssaken)

.... ☐ 1

Q40

	Q40.A Jeg har fulgt rettssaken: Ja (1)	Nei (2)	Q40.B Hvis ja, hvor mye: Noter ant. dager	
Fra en sal med videooverføring	☐	☐	☐	1
Fra hovedforhandlingssalen i Oslo tingrett (sal 250)	☐	☐	☐	2

Q41 **Jeg har vitnet i retten**

Ja .... ☐ 1
Nei .... ☐ 2

Holdninger, tillit og trygghet

Q42 **Nå følger noen spørsmål om sikkerhet, overvåking og åpenhet i samfunnet. Hvor enig eller uenig er du i følgende påstander:**

	Helt enig 1	Delvis enig 2	Verken eller 3	Delvis uenig 4	Helt uenig 5	
• Norsk politi bør begynne å bære våpen	☐	☐	☐	☐	☐	1
• I Norge kan man møte sentrale politikere på gaten uten synlig sikkerhet. Slik bør det fortsatt være.	☐	☐	☐	☐	☐	2
• Sikkerhetstiltakene i Norge bør styrkes, selv om det kan gi ulemper for meg selv.	☐	☐	☐	☐	☐	3

20©12 Ipsos MMI

Q43 **Hvor bekymret er du for nye terrorangrep i Norge i nær framtid?**

Oppgi svaret på en skala fra 0 til 10 der 0 er ikke bekymret i det hele tatt og 10 er svært bekymret?

	Ikke bekymret i det hele tatt										Svært bekymret	
	00	01	02	03	04	05	06	07	08	09	10	
... .	☐	☐	☐	☐	☐	☐	☐	☐	☐	☐	☐	1

Q44 **Hvor stor tillit har du til ...?**

Oppgi på en skala fra 0 til 10 der 0 står for at du ikke har noen tillit i det hele tatt, mens 10 står for at du har full tillit.

	Ingen tillit i det hele tatt										Full tillit	
	00	01	02	03	04	05	06	07	08	09	10	
• Rettsvesenet?	☐	☐	☐	☐	☐	☐	☐	☐	☐	☐	☐	1
• Politiet?	☐	☐	☐	☐	☐	☐	☐	☐	☐	☐	☐	2
• Helsevesenet?	☐	☐	☐	☐	☐	☐	☐	☐	☐	☐	☐	3

20©12 Ipsos MMI

Mestringsstrategier

Q45 **Nå kommer noen spørsmål om ting man kan si eller gjøre for å hjelpe seg selv når noe er vanskelig. Tenk på hva du har pleid å gjøre, eller si til deg selv, i løpet av den siste måneden når du har hatt vansker eller problemer relatert til 22. juli 2011 (for eksempel, søvnvansker, konsentrasjon, sorg).**

Sett ett kryss i hver linje

	Aldri 1	Noen ganger 2	Ofte 3	Meste-parten av tiden 4	
• Jeg tenker på hva som er lurt å gjøre for å håndtere problemet.	☐	☐	☐	☐	1
• Jeg gjør noe for å løse problemet.	☐	☐	☐	☐	2
• Jeg tenker på hva jeg kan lære av det som har skjedd	☐	☐	☐	☐	3
• Jeg prøver å forstå hvordan 22. juli 2011 kunne skje.	☐	☐	☐	☐	4
• Jeg prøver å holde meg unna ting som gjør meg oppskaket.	☐	☐	☐	☐	5
• Jeg snakker med noen som kan hjelpe meg finne ut hva jeg skal gjøre.	☐	☐	☐	☐	6
• Jeg snakker om mine følelser med noen som virkelig forstår	☐	☐	☐	☐	7
• Jeg skylder på andre	☐	☐	☐	☐	8
• Jeg søker trøst i religion.	☐	☐	☐	☐	9
• Jeg sier til meg selv at jeg kan håndtere dette problemet.	☐	☐	☐	☐	10
• Jeg sier til meg selv at ting ville bli bedre.	☐	☐	☐	☐	11
• Jeg minner meg selv på at ting kunne vært verre.	☐	☐	☐	☐	12
• Jeg prøver å la være å tenke på det	☐	☐	☐	☐	13

Q46 **De forrige spørsmålene handlet om hva du gjør når du opplever vanskeligheter relatert til 22. juli 2011. Nå kommer noen påstander om hva du generelt pleier å gjøre når noe er vanskelig. Vennligst sett kryss ved de svarene som passer best for deg.**

Sett ett kryss i hver linje

	Helt galt 1	Nokså galt 2	Nokså riktig 3	Helt riktig 4	
• Jeg klarer alltid å løse vanskelige problemer hvis jeg prøver hardt nok	☐	☐	☐	☐	1
• Hvis noen motarbeider meg, så kan jeg finne måter og veier for å få det som jeg vil	☐	☐	☐	☐	2
• Hvis jeg har et problem og står helt fast, så finner jeg vanligvis en løsning	☐	☐	☐	☐	3
• Jeg føler meg trygg på at jeg vil kunne takle uventede hendelser på en effektiv måte	☐	☐	☐	☐	4
• Jeg beholder roen når jeg møter vanskeligheter, fordi jeg stoler på mine evner til å mestre/få til ting	☐	☐	☐	☐	5

20©12 Ipsos MMI

Livshendelser

Q47 **De neste spørsmålene dreier seg om andre hendelser i livet ditt. Har du noen gang opplevd noen av disse hendelsene?**

Sett ett eller flere kryss for hver linje

	Nei, aldri 1,	Ja, før 22. juli 2011 2,	Ja, etter 22. juli 2011 3.	
• At noen som står deg nær har vært livstruende syk?	☐	☐	☐	1
• Plutselig (uventet) dødsfall hos noen som sto deg nær	☐	☐	☐	2
• En katastrofe (brann, ras, flodbølge, orkan e.l)	☐	☐	☐	3
• En alvorlig ulykke (f.eks. en svært alvorlig bilulykke)	☐	☐	☐	4
• Blitt utsatt for vold (banket opp eller skadet)	☐	☐	☐	5
• Opplevd at noen som stod deg nær bli utsatt for vold (banket opp eller skadet)	☐	☐	☐	6
• Blitt utsatt for ubehagelige seksuelle handlinger (seksuelle overgrep)	☐	☐	☐	7
• Blitt truet eller fysisk/psykisk plaget av medelever/studenter på skolen/studiested i lengere tid	☐	☐	☐	8
• Fått smertefulle eller skremmende behandling på sykehus i forbindelse med sykdom eller skade	☐	☐	☐	9
• Opplevd noe annet som var veldig skremmende, farlig eller voldelig (f.eks. krig, terrorhandlinger, etc.)	☐	☐	☐	10
• Smertefullt brudd med kjæreste/partner, foreldre eller familie	☐	☐	☐	11
• Når barn opplever omsorgssvikt, betyr det at nærstående voksne ikke tar seg så godt av dem som de burde. Det kan for eksempel bety at de ikke gir dem nok mat, tar dem med til legen når de er syke, ordner med rene og varme nok klær eller passer på at de har et trygt sted å være. Har du opplevd omsorgssvikt?	☐	☐	☐	12

Erfaringer med media

Q48 **Har du blitt kontaktet av media om dine erfaringer fra det som skjedde på Utøya 22. juli 2011?**

Ja .... ☐ 1

Nei .... **(⇒ Q51)** ☐ 2

Q49 Hvis ja

Hvordan har du opplevd disse henvendelsene fra media?

Veldig negativt .... ☐ 1

Negativt .... ☐ 2

Både positivt og negativt .... ☐ 3

Positivt .... ☐ 4

Veldig positivt .... ☐ 5

20©12 Ipsos MMI

Q50 **Har du blitt *intervjuet* av media i forbindelse med det som skjedde på Utøya 22. juli 2011?**

Ja .... ☐ 1
Nei .... ☐ 2

Q51 **Har du *skrevet innlegg* i media i forbindelse med det som skjedde på Utøya 22. juli 2011?**

Ja .... ☐ 1
Nei .... ☐ 2

Q52 **Har du blitt intervjuet av media i forbindelse med *rettssaken* etter 22. juli 2011?**

Ja .... ☐ 1
Nei .... ☐ 2

Q53 **Har du skrevet innlegg i media i forbindelse med *rettssaken* etter 22. juli 2011?**

Ja .... ☐ 1
Nei .... ☐ 2

Q54 **Hvis ja på ett eller flere av spørsmålene Q51 - Q53**

	Ikke i det hele tatt	I liten grad	Til dels	I stor grad	I meget stor grad	
	1	2	3	4	5	
• I hvilken grad opplevde du det å være i media som en belastning for deg?	☐	☐	☐	☐	☐	1
• I hvilken grad opplevde du det å være i media som en positiv erfaring for deg?	☐	☐	☐	☐	☐	2
• I hvilken grad har du angret på at du stilte opp i media?	☐	☐	☐	☐	☐	3

Sosiale medier

Du skal svare på disse spørsmålet selv om du tidligere i intervjuet har fortalt om din bruk av sosiale medier. Spørsmålene omhandler all form for kommunikasjon du har hatt gjennom sosiale medier som kan tilknyttes det du opplevde 22. juli og tiden etter.

Q55 **Når du tenker på DEN SISTE MÅNEDEN, har du brukt sosiale medier i tilknytning til det du opplevde 22. juli 2011 og tiden etter?**

Ja .... ☐ 1
Nei ....(⇒ Q60) ☐ 2

Q56 **I løpet av DEN SISTE MÅNEDEN: Hvor ofte har du gjort følgende i tilknytning til det du opplevde 22. juli 2011 og tiden etter?**

Sett ett kryss i hver linje

	Aldri	1-2 ganger i måneden	1-2 ganger i uken	Nesten daglig	Daglig	
	1	2	3	4	5	
• Skrevet/lagt ut/delt noe tilknyttet det du opplevde 22. juli 2011 og tiden etter	☐	☐	☐	☐	☐	1
• Lest om andres tanker eller meninger om hendelsene og tiden etter .	☐	☐	☐	☐	☐	2
• Delt egne tanker eller meninger om det du opplevde og tiden etter ..	☐	☐	☐	☐	☐	3
• Fått støtte fra andre	☐	☐	☐	☐	☐	4
• Gitt støtte til andre	☐	☐	☐	☐	☐	5
• Delt ting du synes det er vanskelig å dele på andre måter	☐	☐	☐	☐	☐	6
• Fortalt andre hvordan du har det	☐	☐	☐	☐	☐	7

20©12 Ipsos MMI

Q57 **I løpet av DEN SISTE MÅNEDEN: Hvem kommuniserer du vanligvis med gjennom sosiale medier i tilknytning til det du opplevde 22. juli 2011 og tiden etter?**

Sett ett kryss

Familie .... ☐ 01
Nære venner/kjæreste/partner (som ikke var på Utøya) .... ☐ 02
Andre som var på Utøya .... ☐ 03

Andre (beskriv): ______________________

____

Q58 **I løpet av DEN SISTE MÅNEDEN: På hvilken måte har du vanligvis kommunisert gjennom sosiale medier i tilknytning til det du opplevde 22. juli 2011 og tiden etter?**

Sett ett kryss

Gjennom åpen kommunikasjon (som hvem som helst kan se) .... ☐ 1
Gjennom lukket kommunikasjon (som kun utvalgte personer kan se) .... ☐ 2
Gjennom både lukket og åpen kommunikasjon .... ☐ 3

Q59 **løpet av DEN SISTE MÅNEDEN: Hvordan har din bruk av sosiale medier i tilknytning til det du opplevde 22. juli 2011 og tiden etter vært for deg?**

Veldig negativt .... ☐ 1
Negativt .... ☐ 2
Både negativt og positivt .... ☐ 3
Positivt .... ☐ 4
Veldig positivt .... ☐ 5

Q60 **Til slutt, helt generelt (uavhengig av hendelsene 22. juli 2011). Hvor mye tid bruker du på sosiale medier en gjennomsnittlig dag?**

0 – 30 minutter .... ☐ 1
31 - 60 minutter .... ☐ 2
Mer enn 60 minutter .... ☐ 3

20©12 Ipsos MMI

Relasjoner/familiesamspill

Q61 **Traumatiske hendelser kan påvirke familielivet. Nedenfor finner du en rekke utsagn som beskriver slike endringer.**

Sett ett kryss i hver linje som indikerer hvor godt hvert utsagn stemmer for deg og din familie.

	Stemmer ikke 0	Stemmer litt 1	Stemmer helt 2	
• Nærmere hverandre enn før	☐	☐	☐	1
• Mer irritable overfor hverandre	☐	☐	☐	2
• Tilbringer mindre tid sammen	☐	☐	☐	3
• Snakker oftere sammen om vanskelige ting	☐	☐	☐	4
• Trekker oss mer unna hverandre	☐	☐	☐	5
• Unngår å diskutere problemer som virker oppskakende	☐	☐	☐	6
• Har mindre kontakt med nære venner og slektninger	☐	☐	☐	7
• Krangler mer med hverandre	☐	☐	☐	8
• Finner det vanskelig å ha glede av felles aktiviteter	☐	☐	☐	9
• Føler at vi har en tydeligere oppfatning om mål og verdier enn før	☐	☐	☐	10
• Bekymrer oss for å legge belastninger på hverandre	☐	☐	☐	11
• Nå etter 22.juli er foreldrene mine mer bekymret for at jeg skal komme til skade	☐	☐	☐	12
• Foreldrene mine har et større behov for å vite hvor jeg er, enn de hadde før 22.juli.	☐	☐	☐	13

Hendelsens betydning

Q62 Tenk på hendelsene på Utøya 22. juli 2011 og besvar hvor enig eller uenig du er i følgende utsagn. Sett ett kryss i hver linje

	Helt enig 1	Delvis enig 2	Verken eller 3	Delvis uenig 4	Helt uenig 5	
• Jeg føler at denne hendelsen har blitt en del av min identitet.	☐	☐	☐	☐	☐	1
• Denne hendelsen har blitt et referansepunkt for hvordan jeg forstår meg selv og verden.	☐	☐	☐	☐	☐	2
• Jeg føler at denne hendelsen har blitt en sentral del av min livshistorie.	☐	☐	☐	☐	☐	3
• Denne hendelsen har farget måten jeg tenker og føler rundt andre opplevelser.	☐	☐	☐	☐	☐	4
• Denne hendelsen har endret livet mitt for alltid.	☐	☐	☐	☐	☐	5
• Jeg tenker ofte på de virkningene denne hendelsen vil ha på min fremtid.	☐	☐	☐	☐	☐	6
• Denne hendelsen var et vendepunkt i livet mitt.	☐	☐	☐	☐	☐	7

©12 Ipsos MMI

Hvordan jeg har tenkt og følt siden det som hendte på Utøya

Q63 **Vi ønsker å få vite hva slags tanker og følelser du har hatt etter det som hendte på Utøya. Nedenfor er en liste med utsagn. Vennligst les hvert utsagn nøye og fortell oss i hvilken grad du er ENIG eller UENIG med hvert utsagn ved å krysse av etter hver setning. Mennesker reagerer på skremmende hendelser på mange ulike måter. Det er ikke noen riktig eller gal måte å svare på disse utsagnene på.**

Sett ett kryss i hver linje

	0 Veldig uenig	1 Litt uenig	2 Litt enig	3 Veldig enig	
	0	1	2	3	
• Hvem som helst kan såre eller skade meg	☐	☐	☐	☐	1
• Alle svikter meg	☐	☐	☐	☐	2
• Jeg er en feiging	☐	☐	☐	☐	3
• Mine reaksjoner etter det som hendte på Utøya betyr at jeg har endret meg til det verre	☐	☐	☐	☐	4
• Jeg stoler ikke på mennesker	☐	☐	☐	☐	5
• Mine reaksjoner etter det som hendte på Utøya betyr at det er noe alvorlig galt med meg	☐	☐	☐	☐	6
• Jeg duger ikke	☐	☐	☐	☐	7
• At jeg ikke klarer å bli kvitt all min redsel betyr at jeg er mislykket	☐	☐	☐	☐	8
• Jeg blir opprørt over småting	☐	☐	☐	☐	9
• Når ting blir vanskelig klarer jeg ikke å håndtere det	☐	☐	☐	☐	10
• Jeg kan ikke hindre at negative ting skjer med meg	☐	☐	☐	☐	11
• Jeg må hele tiden være på vakt for fare	☐	☐	☐	☐	12
• Mine reaksjoner etter det som hendte på Utøya betyr at jeg aldri vil komme over det som skjedde	☐	☐	☐	☐	13
• Før var jeg en glad person, men nå er jeg alltid trist	☐	☐	☐	☐	14
• Det skjer alltid dårlige ting	☐	☐	☐	☐	15
• Jeg kommer aldri til å klare å ha normale følelser igjen	☐	☐	☐	☐	16
• Jeg er redd for at jeg kan bli så sint at jeg kommer til å ødelegge noe eller skade noen	☐	☐	☐	☐	17
• Livet er ikke rettferdig	☐	☐	☐	☐	18
• Livet mitt har blitt ødelagt av det som hendte på Utøya	☐	☐	☐	☐	19
• Jeg føler at jeg er en annerledes person etter det som hendte på Utøya	☐	☐	☐	☐	20
• Mine reaksjoner etter det som hendte på Utøya tyder på at jeg holder på å bli gal	☐	☐	☐	☐	21
• Det kan ikke skje noe bra for meg mer	☐	☐	☐	☐	22
• Noe forferdelig kommer til å skje hvis jeg ikke prøver å kontrollere tankene mine om det som hendte på Utøya	☐	☐	☐	☐	23
• Det som hendte på Utøya har forandret meg for alltid	☐	☐	☐	☐	24
• Jeg må være veldig forsiktig fordi noe fælt kan skje	☐	☐	☐	☐	25

20©12 Ipsos MMI

Positive endringer

Q64 **Noen ganger kan mennesker fortelle om positive endringer etter større traumatiske opplevelser. For hvert av spørsmålene under, indikér i hvilken grad disse forandringene har skjedd i livet ditt som følge av terrorhandlingene 22. juli 2011.**

For hvert av spørsmålene under, indikér i hvilken grad disse forandringene har skjedd i livet ditt som følge av terrorhandlingene 22. juli 2011.

1 = Jeg opplevde ***ikke*** denne forandringen som et resultat av terrorhandlingene.
2 = Jeg opplevde denne forandringen i ***svært liten grad*** som følge av terrorhandlingene
3 = Jeg opplevde denne forandringen i ***liten grad*** som følge av terrorhandlingene
4 = Jeg opplevde denne forandringen i ***middels grad*** som følge av terrorhandlingene
5 = Jeg opplevde denne forandringen i ***stor grad*** som følge av terrorhandlingene
6 = Jeg opplevde denne forandringen i ***veldig stor grad*** som følge av terrorhandlingene

Sett ett kryss i hver linje

	1	2	3	4	5	6	
• Jeg har endret mine prioriteringer når det gjelder hva som er viktig i livet	☐	☐	☐	☐	☐	☐	1
• Jeg setter mer pris på livet mitt	☐	☐	☐	☐	☐	☐	2
• Jeg har fått nye interesser	☐	☐	☐	☐	☐	☐	3
• Jeg har en sterkere følelse av at jeg kan stole på meg selv	☐	☐	☐	☐	☐	☐	4
• Jeg har fått en større forståelse for åndelige ting	☐	☐	☐	☐	☐	☐	5
• Jeg har oppdaget at jeg kan stole på andre i vanskelige perioder	☐	☐	☐	☐	☐	☐	6
• Jeg har lagt om kursen i livet mitt	☐	☐	☐	☐	☐	☐	7
• Jeg føler mer nærhet til andre mennesker	☐	☐	☐	☐	☐	☐	8
• Jeg er mer villig til å uttrykke følelsene mine	☐	☐	☐	☐	☐	☐	9
• Jeg vet nå at jeg kan takle vanskeligheter	☐	☐	☐	☐	☐	☐	10
• Jeg får mer ut av livet mitt nå	☐	☐	☐	☐	☐	☐	11
• Jeg er i større grad i stand til å akseptere ting som de er	☐	☐	☐	☐	☐	☐	12
• Jeg setter mer pris på hver dag som går	☐	☐	☐	☐	☐	☐	13
• Jeg har fått muligheter jeg ellers ikke ville fått	☐	☐	☐	☐	☐	☐	14
• Jeg har fått mer medfølelse for andre	☐	☐	☐	☐	☐	☐	15
• Jeg bestreber meg i større grad på å ivareta mine nære relasjoner	☐	☐	☐	☐	☐	☐	16
• Jeg er mer tilbøyelig til å forandre på ting som trenger å endres	☐	☐	☐	☐	☐	☐	17
• Jeg har en sterkere religiøs tro	☐	☐	☐	☐	☐	☐	18
• Jeg har oppdaget at jeg er sterkere enn jeg trodde jeg var	☐	☐	☐	☐	☐	☐	19
• Jeg har lært mye om hvor flotte mennesker kan være	☐	☐	☐	☐	☐	☐	20
• Jeg aksepterer i større grad at jeg trenger andre	☐	☐	☐	☐	☐	☐	21

20©12 Ipsos MMI

Demografi:

Nedenfor følger noen spørsmål om bosted, familieforhold, inntekt og lignende.

Q65 **Er foreldrene dine skilt/separerte?**

Ja .... ☐ 1
Nei .... ☐ 2

Q66 **Bor du sammen med noen?**

Nei .... ☐ 1
Ja .... ☐ 2

Q67 **Hvis ja, hvem?**
Ett svar for hver linje

	Ja 1	Nei 2	
Ektefelle/samboer	☐	☐	1
En eller begge foreldre	☐	☐	2
Andre voksne	☐	☐	3
Ett eller flere barn (under 18 år)	☐	☐	4

Q68 **Har du flyttet til annen kommune etter 22. juli 2011?**

Ja .... ☐ 1
Nei .... ☐ 2

Q69 **Er du i arbeid (hel- eller deltid, minst 50%)?**

Ja .... ☐ 1
Nei .... ☐ 2

Q70 **Er du student eller skoleelev (hel- eller deltid, minst 50%)?**

Ja .... ☐ 1
Nei .... ☐ 2

HVIS JA PÅ SPM. **Q69**

Q71 **Har du vært borte fra jobb på grunn av fysiske eller psykiske plager i løpet av de siste 3 månedene?**

Nei .... ☐ 1
Ja .... ☐ 2

HVIS JA PÅ SPM. **Q70**

Q72 **Har du vært borte fra skole på grunn av fysiske eller psykiske plager i løpet av de siste 3 månedene?**

Nei .... ☐ 1
Ja .... ☐ 2

HVIS JA PÅ SPM. **Q71** ELLER **Q72**

Q73 **Omtrent hvor mange dager med sykefravær har du hatt til sammen de siste 3 månedene?**

5 dager eller mindre .... ☐ 1
6-10 dager .... ☐ 2
11-20 dager .... ☐ 3
Mer enn 20 dager .... ☐ 4

Q74 **Var du med på forrige intervjurunde i studien?**

Ja ....(⇒ **Q91**) ☐ 1
Nei .... ☐ 2

TIL DEG SOM IKKE VAR MED PÅ FORRIGE INTERVJURUNDE

Q75 **Kjønn**

Kvinne/jente .... ☐ 1
Mann/gutt .... ☐ 2

Q76 **Antall søsken (inklusive halvsøsken og stesøsken)**

Noter antall ☐ 1

Q77 **Antall foreldre som er i live (biologiske og adoptivforeldre)**

0 .... ☐ 0
1 .... ☐ 1
2 .... ☐ 2

Q78 **Er du født i Norge?**

Ja .... ☐ 1
Nei .... ☐ 2

HVIS FØDT I ANNET LAND

Q79 **Hvilket land ble du født i?**

Noter ______________________________

____

Q80 **Er moren din født i Norge?**

Ja .... ☐ 1
Nei .... ☐ 2

20©12 Ipsos MMI

HVIS MOREN DIN BLE FØDT I ANNET LAND

Q81 **Hvilket land ble hun født i?**

Noter ______________________________

____

Q82 **Er faren din født i Norge?**

Ja .... ☐ 1
Nei .... ☐ 2

HVIS FAREN DIN BLE FØDT I ANNET LAND

Q83 **Hvilket land ble han født i?**

Noter ______________________________

____

Q84 **Hva er din høyeste fullførte utdanning/skolegang?**

Universitet/høyskole mer enn 4 år .... ☐ 1
Universitet /høyskole inntil 4 år .... ☐ 2
Studieforberedende opplæring / allmennfaglig studieretning på videregående skole .... ☐ 3
Yrkesfaglig opplæring / yrkesfaglig studieretning på videregående skole .... ☐ 4
Grunnskole .... ☐ 5
Ingen fullført utdannelse .... ☐ 6

TIL DEM SOM BOR MED FORELDRE

Q85 **Hvor god råd synes du at familien din har i forhold til folk flest?**

Mye bedre råd .... ☐ 1
Noe bedre råd .... ☐ 2
Omtrent som folk flest .... ☐ 3
Noe dårligere råd .... ☐ 4
Mye dårligere råd .... ☐ 5

TIL DEM SOM IKKE BOR MED FORELDRE

Q86 **Hvor god råd synes du at du har i forhold til folk flest?**

Mye bedre råd .... ☐ 1
Noe bedre råd .... ☐ 2
Omtrent som folk flest .... ☐ 3
Noe dårligere råd .... ☐ 4
Mye dårligere råd .... ☐ 5

Q87 **Var du medlem i AUF 22. juli 2011?**

Ja .... ☐ 1
Nei .... ☐ 2

Q88 **Er du medlem i statskirken eller annet kristent trossamfunn?**

Ja .... ☐ 1
Nei .... ☐ 2

Q89 **Er du medlem av islamsk trossamfunn?**

Ja .... ☐ 1
Nei .... ☐ 2

Q90 **Er du medlem av noen andre trossamfunn?**

Ja .... ☐ 1
Nei .... ☐ 2

20©12 Ipsos MMI

Hjelpetiltak

Q91

	**Q91.A** Har du hatt kontakt med noen av følgende personer/instanser inneværende år (2012)?		**Q91.B** Hvis ja, ca hvor mange ganger?				**Q91.C** Hvis ja, i hvilken grad har denne hjelpen vært til nytte for deg?			
	**Ja**	**Nei**	**Ingen timer**	**1-3 timer**	**4-10 timer**	**11 timer eller flere**	**Ikke i det hele tatt**	**I noen grad**	**I stor grad**	
	1	2	1	2	3	4	1	2	3	
• **Kontaktpersonen du fikk oppnevnt rett etter hendelsen**	☐	☐	☐	☐	☐	☐	☐	☐	☐	1
• **Fastlegen din**	☐	☐	☐	☐	☐	☐	☐	☐	☐	2
• **Andre hjelpepersoner i kommunen (både i helsetjenesten, psykisk helsevern eller andre kommunale tjenester)** . .	☐	☐	☐	☐	☐	☐	☐	☐	☐	3
• **Spesialister (som psykologer/psykiatere på sykehus, poliklinikk, privat el.l)**	☐	☐	☐	☐	☐	☐	☐	☐	☐	4
• **Sjelesørger (prest, imam)**	☐	☐	☐	☐	☐	☐	☐	☐	☐	5

Psykisk helse før terroren

Q92 **Har du noen gang, før 22. juli 2011. . .**

	Ja	Nei	
	1	2	
• Fått hjelp fra psykisk helsevesen (for eksempel BUP, DPS, privatpraktiserende psykolog/psykiater)?	☐	☐	1
• Fått hjelp fra andre instanser (for eksempel PPT, familievernkontor, barneverntjenesten)? . . .	☐	☐	2
• Fått medikamenter mot depresjon, angst eller andre psykiske problemer?	☐	☐	3
• Kjent deg plaget av å være nedstemt, deprimert eller følelse av håpløshet i fire uker eller mer?	☐	☐	4
• Vært plaget av panikkfølelser eller vedvarende uro eller angst i fire uker eller mer?	☐	☐	5

Når du har fylt inn spørsmålene ovenfor, vil intervjuer avslutte med å diskutere hjelpebehov med deg.

20©12 Ipsos MMI

Del IV Vurdering av udekket hjelpebehov

Udekket hjelpebehov

Spørsmålene nedenfor fylles ut av intervjuer i samråd med deg

Q93

Instruksjon til intervjuer:
Hvis informanten svarte litt eller mer plaget på spørsmål om «Tanker om å ta ditt liv» (på Q6) foretas en suicidalitetsvurdering hvor blant annet følgende momenter bør dekkes:

	Ja 1	Nei 2	
Har du noen gang skadet deg selv?	☐	☐	1
Har du skadet deg selv i løpet av de siste 14 dagene?	☐	☐	2
Har du konkrete tanker om å ta ditt eget liv?	☐	☐	3
Har du planer om å ta ditt eget liv?	☐	☐	4

FOR ALLE

Q94 **Får du dekket de behovene du mener du har for hjelp nå (for eksempel fra psykolog, lege, sykepleier, sosial arbeider, eller andre profesjonelle)?**

Ja ☐ 1
Nei ☐ 2

Instruksjon til intervjuer:
På bakgrunn av svar på spørsmål **Q93** og **Q94** skal det foretas en klinisk vurdering av hjelpebehov. Hvis det er udekket hjelpebehov skal intervjuer følge opp dette. Oppfølgingen kan være formidling av kontakt med hjelpeapparatet eller informasjon om hvordan få nødvendig hjelp. Ved behov kan det tilbys telefonisk oppfølging fra deg for å høre om personen har fått kontakt med hjelpeinstans.

Q95 **Er det i orden at vi kontakter deg igjen om 1,5 år for et nytt intervju?**

Ja ☐ 1
Nei ☐ 2

Avslutning av intervjuet

Instruksjon til intervjuer:

Gi deltakeren god tid til å avslutte intervjuet eller kommentere. Kanskje er det ting de ønsker du skulle ha spurt om eller annet de ønsker å formidle tilbake til oss. Dersom deltakeren trenger tid til å avslutte, vis respekt for det. Hjelp til med eventuelle emosjonelle reaksjoner som de trenger hjelp til. Husk å takke for at de har deltatt og minn dem på at all kontaktinformasjon står i informasjonsskrivet.

20©12 Ipsos MMI
